# *GZMK* expression within activated intratumoral T-cell subsets reflects differentiation efficiency and predicts response to cancer immunotherapy

**DOI:** 10.1038/s41698-026-01437-7

**Published:** 2026-04-18

**Authors:** Cem M. Sievers, Tian-Gen Chang, Yvette Robbins, Jay Friedman, Marco Craveiro, Xinping Yang, Christopher Silvin, Jason M. Redman, Patrick Soon-Shiong, Wiem Lassoued, Martha Quezado, Wojciech Mydlarz, Nancy P. Judd, Jeffrey Schlom, James Gulley, Eytan Ruppin, Clint T. Allen

**Affiliations:** 1 Head and Neck Section, Surgical Oncology Program, Center for Cancer Research, National Cancer Institute, National Institutes of Health, Bethesda, MD USA; 2 Bioinformatics Section, Surgical Oncology Program, Center for Cancer Research, National Cancer Institute, National Institutes of Health, Bethesda, MD USA; 3 Cancer Data Science Laboratory, Center for Cancer Research, National Cancer Institute (NCI), National Institutes of Health (NIH), Bethesda, MD USA; 4 Center for Immuno-Oncology, Center for Cancer Research, National Cancer Institute, National Institutes of Health, Bethesda, MD USA; 5 ImmunityBio, Culver City, CA USA; 6 Laboratory of Pathology, Center for Cancer Research, National Cancer Institute, National Institutes of Health, Bethesda, MD USA; 7 Department of Otolaryngology-Head and Neck Surgery, Johns Hopkins School of Medicine, Baltimore, MD USA; 8 Kiaser Permanente of Northern Virginia, Alexandria, VA USA

**Keywords:** Cancer, Immunology, Oncology

## Abstract

Neoadjuvant immunotherapies (NITs) have demonstrated clinical benefit in head and neck carcinoma and other cancers by enhancing T cell-mediated anti-tumor immunity. However, disease recurrence remains a major challenge in a significant proportion of patients. Characterization of T cell dynamics underlying NIT outcomes may lead to improved treatment strategies. Here, we identify baseline intratumoral T cell differentiation efficiency as a predictor of response to NIT. Clonal analysis of tumor-emergent T cells post-treatment revealed granzyme K (*GZMK)* expression within differentiated subsets as a marker of recent differentiation. Efficient pre-treatment differentiation of *GZMK*^+^ progenitor T cells toward activated effectors predicts increased treatment-induced tumor regression. Consistently, pre-treatment tumor-infiltrating T cell clones predominantly adopt either exhausted, tissue-resident memory-like, or peripherally enriched *GZMK*^+^ progenitor states, implicating impaired intratumoral differentiation in limited anti-tumor immunity at baseline. Together, our findings demonstrate that *GZMK*^*+*^ T cell profiles reflect baseline anti-tumor immunocompetence and offer a clinically actionable biomarker for predicting immunotherapy response. NCT04247282, ClinicalTrails.gov, registered 1/30/2020 and NCT03429036, ClinicalTrails.gov, registered 11/06/2020.

## Introduction

T cell-based cancer immunotherapies, including immune checkpoint blockade (ICB), have substantially lowered recurrence rates in head and neck squamous cell carcinomas (HNSCC) and other solid tumors^1–5^. Pathologic response, defined as the extent of treatment-induced tumor regression within the resected tumor bed, serves as a direct measure of neoadjuvant immunotherapy (NIT) efficacy. Notably, higher rates of pathologic response have been consistently associated with improved recurrence-free survival across multiple cancers^6,7^. Predictive biomarkers of pathologic response to NIT remain incompletely characterized.

Anti-tumor immunity is orchestrated through a series of coordinated steps involving the activation of tumor-specific T cells in the tumor-draining lymph nodes, followed by their trafficking through the peripheral circulation to the tumor site^8,9^. Upon tumor entry, conditions within the tumor microenvironment (TME) drive phenotypic transitions within T cells^10^. Chronic T cell receptor (TCR) stimulation, resulting from persistent tumor-antigen exposure^11–14^, can induce T cell exhaustion characterized by the expression of inhibitory receptors such as PD1, LAG3, and TIM3. Exhausted T cells also display features of tissue residency and exhibit reduced levels in peripheral blood^14^. In contrast, tumor-specific T cells in circulation display reduced expression of exhaustion markers and frequently adopt progenitor central memory or granzyme K–positive effector-memory T cell (*GZMK*^+^ Tem) phenotypes^15^. The presence of different T cell subsets within tumors has been associated with response to immunotherapy^16–19^. For instance, increased frequency of *TCF7*^+^ progenitor T cells was associated with successful ICB immunotherapy in patients with relapsed cancer^18^. Similarly, reactivation of exhausted, tissue-resident T cells has been linked to pathologic response following NIT^16,17^. Notably, tumor-specific T cell clones can exist across a range of phenotypic states. Despite these observations, how developmental trajectories within the intratumoral T cell compartment relate to NIT treatment response is less well understood.

In this study, we performed a comprehensive clonal and phenotypic analysis of tumor-infiltrating T lymphocytes (TILs) using combined single-cell RNA and TCR sequencing as well as protein-level analysis across pre- and post-treatment time points in patients receiving NIT. We demonstrate that *GZMK* expression can be used to characterize differentiation processes of peripherally derived *GZMK*^+^ Tem cells within tumors, linking *GZMK* expression to recent tumor entry and activation potential. Our findings uncover key treatment-induced changes in T cell developmental hierarchies and identify enhanced baseline activation and differentiation of tumor-infiltrating *GZMK*^+^ Tem cells as a defining feature of elevated immunocompetence and improved NIT outcomes.

## Results

### Phenotypic characterization of tumor-infiltrating CD4^+^ and CD8^+^ T cells

**Fig. 1:**
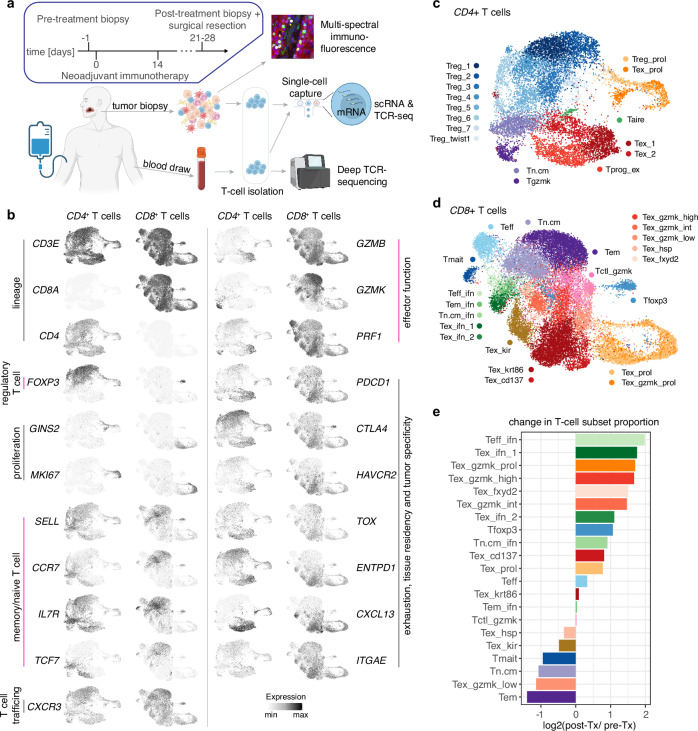
Phenotypic characterization of tumor-infiltrating CD4+ and CD8+ T cells. **a** Schematic illustrating the data-generating process. Created in BioRender (https://BioRender.com/ze97jrj). **b** Scatter plot showing uniform manifold approximation and projection (UMAP) embedding of *CD4*^+^ and *CD8*^+^ tumor-infiltrating T cells colored by expression of indicated genes grouped into different functional categories. Scatter plots showing UMAP embedding of tumor-infiltrating **c** *CD4*^+^ - and **d** *CD8*^+^ T cells colored by inferred T cell identity. **e** Bar graph showing the log2-transformed change in proportion, comparing post- to pre-Tx proportions, associated with different *CD8*^+^ T cell phenotypes.

To study molecular mechanisms underlying responses to T cell-based immunotherapies, we isolated T cells from pre- and post-treatment tumor biopsies of eleven patients with newly diagnosed, HPV-negative HNSCC receiving NIT, using a bispecific inhibitor of TGF-β and PD-L1 alone or in combination with a therapeutic vaccine targeting tumor-associated antigens, as previously described in refs. ^4,20^ (Tables S1 and S2). Isolated T cells underwent combined single-cell RNA and V(D)J-sequencing (scRNA/VDJ-seq; Fig. 1a; Table S2). Of note, the current study expands upon a previously published analysis of pre- and post-treatment (pre- and post-Tx) data from six patients treated with the bispecific PD-L1 and TGF-β inhibitor as monotherapy^14^ to include paired data from five new patients treated with combination therapy (Table S2). Considering the limited sample size per treatment arm, we performed a combined analysis of pre- and post-Tx samples. In total, we obtained high-quality expression data from 79,836 tumor-infiltrating T cells from paired pre- and post-Tx samples of eleven patients, of which 60,978 cells were assigned to 31,964 distinct TCR clonotypes (Fig. S1a). Clonotype-level analysis based on *CD4* and *CD8* expression identified 12,504 *CD4*^+^ and 25,092 *CD8*^+^ T cells, respectively (Fig. S1b–e). Graph-based clustering and marker gene expression profiling revealed distinct T cell subsets (Fig. 1b–d).

Among the *CD4*^+^ T cells, Treg clusters were defined by expression of *FOXP3*, *IL2RA*, and *CTLA4* (Fig. 1b, c; Fig. S1f). Other T cells were characterized by expression of *CCR7*, *IL7R,* and *TCF7* and may be composed of both naïve and central-memory phenotypes (Tn.cm) given their transcriptional similarity^21^ (Fig. 1b, c; Fig. S1f). We also observed T cells that expressed relatively high levels of effector and exhaustion genes (Tex). Of note, T cell exhaustion can result from chronic TCR stimulation and has been associated with tumor specificity^12–14^. In addition, relatively high levels of exhaustion markers were also observed in a progenitor population (Tprog_ex; Fig. 1b, c; Fig. S1f). We also observed a relatively small population of extrathymic AIRE-expressing *CD4*^+^ T cells^22^ (Taire), enriched in a single sample and characterized by relatively high HLA class II (Fig. S1f), possibly indicating a role in antigen presentation as recently described^23^. The proliferative *CD4*^+^ compartment was mostly composed of exhausted (Tex_prol) or regulatory T cells (Treg_prol; Fig. 1b, c; Fig. S1f).

In contrast to *CD4*^+^ T cells, *CD8*^+^ T cells were characterized by an increased proportion of Tex cells that displayed considerable transcriptional heterogeneity (Fig. 1b, d, Fig. S1g). For instance, subsets of Tex cells expressed relatively high levels of *KRT86*, which has been associated with a tissue-resident phenotype^24,25^ (Tex_krt86), the co-stimulatory receptor *CD137*/4-1BB (Tex_cd137), or heat shock genes (Tex_hsp). Notably, *KRT86* and *CD137* expression in T cells has previously been linked to tumor-reactivity^24–26^. KIR-expressing *CD8*^+^ T cells (Tex_kir) also displayed increased expression of γδTCR genes (Fig. S1h), suggesting that this population may harbor a number of γδ T cells, as has been observed by others^27^. *CD8*^+^ Tex cells displayed increased expression of genes associated with tissue residency, such as *ITGAE* and *ZNF683* (Fig.1b; Fig. S1g). Additional *CD8*^+^ T subsets included Teff cells, expressing various effector genes in addition to *CX3CR1*, mucosal-associated invariant T cells (Tmait), and a distinct population characterized by *FOXP3* expression (Tfoxp3; Fig. S1g, i). We also observed a distinct cluster composed of different T cell subsets that shared common expression of IFNγ-response genes, possibly indicative of locally elevated IFNγ levels (Fig. 1b, d; Fig. S1g,j). Similar to the *CD4*^+^ T cell compartment, we identified different *CD8*^+^ memory T cell subsets, including Tn.cm cells and an effector-memory-like (Tem) population characterized by increased *GZMK* expression. Notably, *GZMK* expression was not restricted to Tem cells, but also detectable in different Tex clusters to varying degrees (Fig. 1b, d; Fig. S1g). For instance, within the proliferative compartment (Tex_prol), we observed a subset of cells that differed based on the expression of *GZMK* (Tex_prol_gzmk), suggesting that *GZMK*^+^ cells may undergo activation-induced proliferation within tumors. Despite comparable expression of exhaustion and effector genes, CD8^+^ Tex subsets exhibited significant differences in transcriptional activity related to various functional gene sets associated with, for instance, T cell function, proliferation, metabolism, immunity, or interferon signaling (Fig. S2, Table S3). Furthermore, Tex subsets displayed substantially increased expression of the NeoTCR signature, derived from neoantigen-specific T cells^11^ (Fig. S3a, b). We also observed a significant correlation between T cell exhaustion and the NeoTCR8 signature, further supporting the idea that, within the TME, the exhausted T cell compartment is enriched in tumor-specific T cells (Fig. S3c).

All major *CD4*^+^ and *CD8*^+^ T cell subsets were present across samples (Fig. S3d, e). Furthermore, compared to pre-Tx conditions, post-Tx *CD8*^+^ T cells exhibited reduced Tem proportions and a concomitant expansion of different *GZMK*^+^ Tex phenotypes (Fig. 1e, Fig. S3f, g). These findings reveal substantial phenotypic variability within tumor-infiltrating T cells and suggest treatment-induced differentiation of *GZMK*^+^ Tem progenitors.

### Phenotype co-occurrence analysis within individual T cell clones indicates limited differentiation prior to NIT

**Fig. 2:**
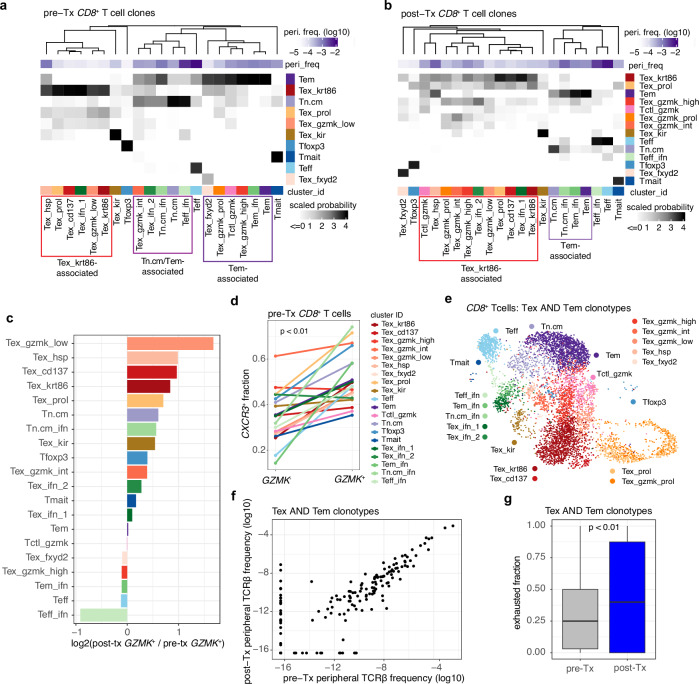
Phenotypic compartmentalization within CD8+ TIL is reduced following treatment. Heatmaps showing column-standardized, weighted averages of probabilities of two phenotypes co-occurring in a *CD8*^+^ T cell clone in both **a** pre-Tx and **b** post-Tx T cells. Clone-based probability estimates were multiplied by the normalized log10-transformed clone size to account for differences in clone size. The average log10-transformed peripheral TCRβ frequencies of the associated clonotypes are shown above. **c** Bar graph showing the log2-transformed fold change in the fraction of *GZMK*-positive pre- and post-Tx T cells for different *CD8*^+^ T cell phenotypes. **d** Line graph showing the fraction of *CXCR3*-positive pre-Tx *CD8*^+^ T cells associated with different phenotypes that are distinguished based on the presence or absence of GZMK expression. *P*-value; paired Wilcoxon rank-sum test. **e** Scatter plots showing UMAP embedding of *CD8*^+^ T cells associated with at least one Tem cell and one Tex cell, colored by T cell phenotype. **f** Scatter plot showing pre- and post-Tx log10-transformed TCRβ frequencies of Tex/Tem clonotypes shown in (**e**). **g** Box plot showing the pre- and post-Tx fraction of exhausted T cells associated with Tem/Tex clonotypes. *P*-value; Wilcoxon rank-sum test.

To explore T cell development underlying phenotypic diversity of tumor-infiltrating T cells at the clonal level, we evaluated phenotype co-occurrence within individual TCR-defined clones. Both pre- and post-Tx *CD4*^+^ T cell clones were largely compartmentalized into Treg and non-Treg phenotypes, suggesting a relatively high phenotypic consistency within individual clones (Fig. S4a,b). Similarly, CD8+ Tex_kir, Tfoxp, Tmait, and Teff cells displayed high phenotypic consistency, indicating that these cells have limited developmental plasticity (Fig. 2a, b).

Further analysis of pre-Tx CD8+ T cell clones revealed three main clusters of co-occurring phenotypes defined by the predominant association with either Tex_krt86 (Tex_krt86-associated), both Tn.cm and Tem (Tn.cm/Tem-associated), or Tem alone (Tem-associated) cells (Fig. 2a). The Tex_krt86-associated cluster was enriched in differentiated and exhausted T cell subsets and included Tex_prol, Tex_cd137 as well as Tex_gzmk_low T cell phenotypes. In contrast, the Tn.cm/Tem-associated cluster was associated with Tex_gzmk_int but generally showed reduced co-occurrence with exhausted phenotypes. The Tem-associated cluster displayed increased phenotypic association with GZMK-positive, exhausted phenotypes, such as Tex_gzmk_prol, Tct_gzmk, Tex_gzmk_hi, and Tex_fxyd2 cells, potentially reflecting early differentiation of Tem toward more differentiated T cell subsets that still retain detectable *GZMK* transcript levels. These findings suggest that prior to NIT, *CD8* + T cell clones are largely confined to distinct phenotypic compartments.

### TILs display expression changes indicative of increased differentiation of GZMK+ progenitors following NIT

In contrast to pre-Tx, post-Tx *CD8*^+^ T cells displayed profound reorganization related to phenotypic compartments indicative of greater phenotypic diversity within distinct T cell clones (Fig. 2b). While the Tn.cm/Tem-associated cluster was absent post-Tx, the Tem-associated cluster was smaller and displayed reduced associations with exhausted phenotypes. In contrast, the Tex_krt86-associated cluster was expanded by additional phenotypes. Interestingly, many of these novel post-Tx phenotypic associations of the Tex_krt86-associated cluster involved *GZMK*^+^ Tex phenotypes, such as Tex_gzmk_prol, Tctl_gzmk, and Tex_gzmk_high, that were previously associated with Tem, potentially indicating an increased differentiation of *GZMK*^+^ progenitors toward more terminally differentiated phenotypes after NIT. In agreement with a treatment-induced increase in differentiation of *GZMK*^+^ progenitors, various Tex clusters displayed an elevated proportion of *GZMK*^+^ cells post-Tx (Fig. 2c). A similar trend was also observed in C*D4*^+^ T cells (Fig. S4c), suggesting that differentiation of *GZMK*^+^ progenitors is not restricted to the CD8 compartment^28^. To rule out that G*ZMK* expression differences resulted from technical artifacts such as batch correction, we evaluated T cell marker gene expression in distinct T cell phenotypes partitioned into *GZMK*^+^ and *GZMK*^-^ subsets. This analysis revealed a substantial transcriptional similarity across individual T cell phenotypes comparing *GZMK*^+^ and *GZMK*^-^ subsets, suggesting that different T cell phenotypes can form regardless of *GZMK* expression (Fig. S4d).

*GZMK*^+^*CD8*^+^ Tem cells are prevalent in healthy human blood samples^29^. Consistent with the recent emergence from circulation in the tumor, *GZMK*-positivity was associated with increased expression of *CXCR3*, a chemokine receptor that mediates T cell chemotaxis, across a range of *CD8*^+^ T cell phenotypes (Fig. 2d; Fig. S4e–g).

To further explore the distribution of distinct T cell clones across anatomical sites before and after NIT, we evaluated peripheral T cell clonotype frequencies within pre- and post-Tx peripheral-blood samples using deep TCRβ sequencing (Table S2). Consistent with the increased expression of tissue residency markers, various Tex subsets associated with the Tex_krt86-associated cluster were characterized by reduced frequencies in pre-Tx blood, suggesting that the corresponding T cell clones exhibit relative depletion from circulation (Fig. 2a). In contrast, the Tn.cm/Tem- and Tem-associated clusters displayed increased peripheral frequencies, indicative of expansion in the peripheral blood (Fig. 2a). Previous work has demonstrated that neoadjuvant immunotherapies can lead to an increase of circulating tumor-specific Tex cells post-Tx^14,16,30,31^. Consistent with our prior observations, clonotypes associated with various Tex phenotypes displayed increased peripheral frequencies post-Tx (Fig. 2a, b; Fig. S4h). This post-Tx peripheral increase of Tex cells can, for instance, be explained by a treatment-induced reduction of tissue retention and a subsequent egress of Tex cells from the tumor into circulation^14,27,28^. Consistent with this mechanism, TCRβ frequencies of various *CD8*^+^ Tex cells that were detected within pre-Tx tumors displayed a significant increase in post-Tx blood samples (Fig. S4i).

In addition, increased treatment-induced differentiation of progenitor populations that display relatively high peripheral frequencies prior to treatment, such as Tem cells, toward exhausted T cell subsets may constitute an alternative mechanism contributing to the enhanced post-Tx clonotype-mediated association between the exhausted, intratumoral T cell compartment and peripheral circulation (Fig. 2a, b; Fig. S4h). To explore this possibility, we focused on hybrid *CD8*^+^ T cell clonotypes defined as individual T cell clones harboring at least one exhausted and one Tem cell (Fig. 2e). Notably, these hybrid clonotypes did not display significant differences between pre- and post-Tx peripheral frequencies (Fig. 2f; Fig. S4j). However, the proportion of exhausted cells per clonotype was increased post-Tx, suggesting that enhanced treatment-induced differentiation of frequent peripheral T cell clones also contributes to the emergence of Tex cells that maintain relatively high levels in circulation (Fig. 2g).

In summary, these data are consistent with increased activation and differentiation of *GZMK*^+^ progenitor T cells following NIT that traffic from the periphery into tumors to span the intratumoral Tex and peripheral-blood compartments.

### Distribution of *CD8*^+^ Tex clonotypes across tumor and blood identifies *GZMK* expression as a marker of recent tumor infiltration

**Fig. 3:**
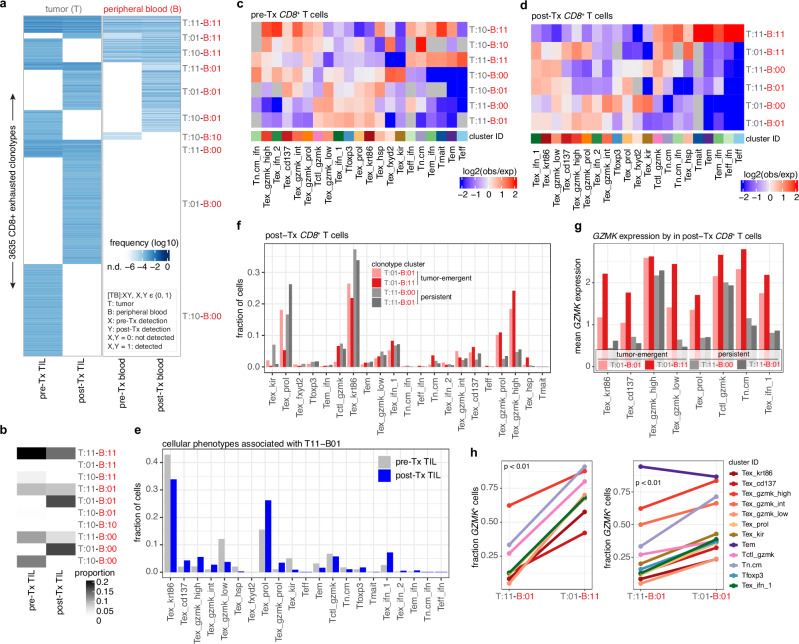
Emergence from peripheral blood is associated with GZMK expression. **a** Heatmap showing intratumoral and peripheral TCRβ frequencies pre- and post-Tx of *CD8*^+^ T cell clonotypes associated with at least one Tex cell. Pre-Tx TIL frequencies represent a combination of scRNA-seq- and deep tumor TCRβ sequencing-based frequencies, which were available for pre-Tx samples only. **b** Heatmap showing the proportion of pre- and post-Tx *CD8*^+^ T cells associated with the indicated clonotype classes derived from (**a**). Heatmaps showing the phenotypes associated with distinct clonotype classes quantified by comparing observed and expected frequencies within **c** pre- and **d** post-Tx tumor-infiltrating *CD8*^+^ T cells. **e** Bar graph showing the fraction of pre- and post-Tx *CD8*^+^ T cells associated with the T:11-B:01 clonotype class assigned to indicated phenotypes. Bar graph showing the **f** fraction or **g** *GZMK* expression of pre- and post-Tx T cells associated with either tumor-emergent clonotype classes (T:01-B:01 and T:01-B:11) or pre-Tx tumor confined clonotype classes (T:11-B:00 and T:11-B:01) assigned to indicated phenotypes. **h** Line graphs showing the fraction of *GZMK*-positive post-Tx *CD8*^+^ T cells within the pre-Tx tumor confined clonotype class T:11-B:01 and the tumor-emergent clonotype classes (left) T:01-B:11 or (right) T:01-B:01 for the indicated phenotypes.

To study the dynamics of T cells enriched for tumor specificity across distinct anatomical compartments, we focused on *CD8*^+^ T cell clonotypes associated with exhausted cells and evaluated their frequencies in pre- and post-Tx tumor and peripheral-blood samples. This analysis revealed several clonotype classes that displayed different frequency patterns across treatment states and anatomical sites (Fig. 3a). For instance, some clonotypes were detected in pre- and post-Tx tumor and blood (T:11-B:11), while others were absent in pre-Tx blood (T:11-B:01). Notably, persistent T cell clonotypes, detected in both pre- and post-Tx tumor samples, were generally expanded within the tumor (Fig. S5a).

For each clonotype class, we next quantified the relative contribution to the *CD8*^+^ TIL compartment and their associated phenotypes (Fig. 3b–d). T:11-B:11 clonotypes, detected in the tumor and blood pre- and post-Tx, constituted a substantial proportion of both pre- and post-Tx *CD8*^+^ TILs (Fig. 3b) and were enriched in Tem and other memory/progenitor phenotypes (Fig. 3c, d; Fig. S5b). In contrast, although undetected in the blood, T:10-B:00- and T:01-B:00 – associated T cells also displayed a relatively high abundance (Fig. 3b) but were enriched in Tex_kir and Tex_fxyd2 phenotypes (Fig. 3c, d). Persistent TIL clonotypes that emerged in the post-Tx blood (T:11-B:01) were enriched in different exhausted phenotypes (Fig. 3c, d, Fig. S4b) and may correspond to T cells that undergo treatment-induced activation and subsequent tumor egress; supporting this notion, we observed a decrease in Tex_krt86 cells and a concomitant increase in Tex_prol cells after treatment within these clonotypes (Fig. 3e). Furthermore, the exhausted T:11-B:01 T cell compartment was characterized by a significant reduction in the tissue-residency marker *ITGAE* (encoding CD103), consistent with previously described mechanisms of decreased tissue residency after immunotherapy through reduced expression of CD103^14^ (Fig. S5c).

We then compared clonotypes that emerged within post-Tx tumors, possibly from the blood (T:01-B:01 and T:01-B:11), to non-emergent/persistent, tissue-resident clonotypes that were either not detectable in the blood at all (T:11-B:00) or possibly emerged in the blood from the tumor post-Tx (T:11-B:01). Although the persistent clonotypes were enriched in the Tex_krt86 phenotype, the tumor-emergent clonotypes displayed greater association with *GZMK*^+^ expressing phenotypes Tex_gzmk_prol and Tex_gzmk_high (Fig. 3f). Furthermore, we observed elevated *GZMK* expression in tumor-emergent clonotypes across a range of T cell phenotypes (Fig. 3g, h; Fig. S5d, e), suggesting that *GZMK* expression marks emergent T cells and recent intratumoral differentiation.

### Activation of peripheral-blood Tem cells leads to decreased *GZMK* expression and acquisition of Tex features

**Fig. 4:**
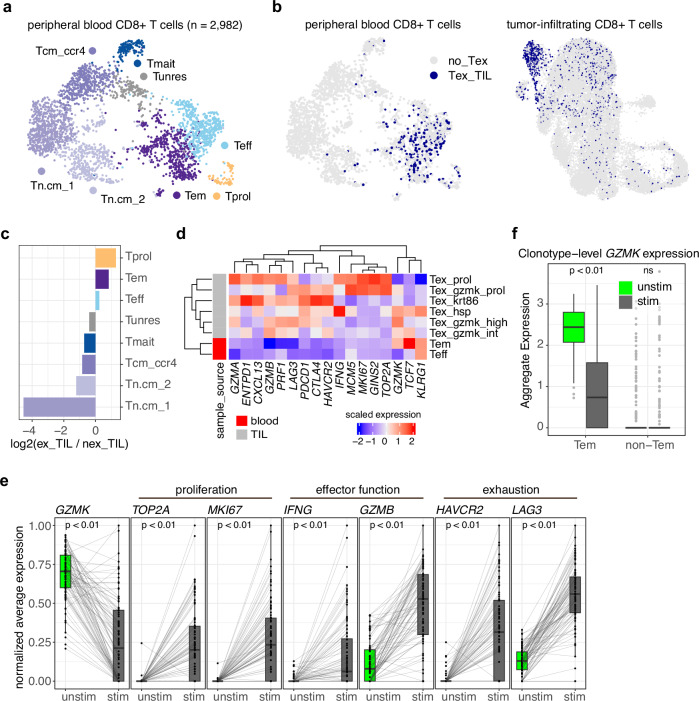
Peripheral-blood GZMK+ Tem cells undergo Tex phenotype transition upon TCR stimulation. **a** Scatter plots showing UMAP embedding of patient-matched post-Tx peripheral-blood *CD8*^+^ T cells colored by inferred phenotype isolated from eleven patients with available TIL scRNA/VDJ-seq. **b** Scatter plots showing UMAP embedding of post-Tx peripheral-blood *CD8*^+^ T cells (left) and TIL (right) obtained from the same patients. Color distinguishes all T cells based on the presence of exhausted TIL within corresponding T cell clones; in total, 139 clonotypes that meet these requirements were identified. **c** Bar plot comparing phenotype proportions of peripheral-blood *CD8*^+^ T cells clonally associated with either at least one exhausted TIL or only non-exhausted TILs. The corresponding phenotype proportions are compared, considering the log2-transformed ratio. **d** Heatmap showing column-standardized average expression of T cell marker genes within the indicated peripheral-blood and tumor-infiltrating T cell subsets. Only T cells clonally associated with at least one exhausted TIL are considered. **e** Box plots showing clonotype-average gene expression of peripheral-blood-derived *CD8*^+^ T cells associated with Tem clonotypes, i.e., all clonotypes with at least two Tem cells and non-zero GZMK expression, comparing unstimulated and TCR-stimulated T cells. Only clonotypes shared between experimental conditions were considered. *P*-value; paired Wilcoxon rank-sum test. **f** Box plot showing clonotype-averaged *GZMK* expression within blood-derived *CD8*^+^ T cells, obtained from four additional treatment-naïve patients, classified as Tem clonotypes or non-Tem clonotypes, i.e., all clonotypes that contain no Tem cells, comparing unstimulated and TCR-stimulated T cells. Only clonotypes shared between experimental conditions were considered in this analysis. *P*-value; paired Wilcoxon rank-sum test.

To further study differentiation processes, in addition to the TIL data, we generated scRNA/VDJ-seq data of T cells isolated from patient-matched post-Tx peripheral-blood (PB) samples (Fig. 4a, Table S2). Within the PB *CD8*^+^ compartment, we identified various T cell phenotypes consistent with naïve/memory, effector, MAIT, or proliferating T cell subsets (Fig. 4a, Fig. S6a–c), in agreement with previous studies^29,32,33^. Compared to *CD8*^+^ TIL, peripheral T cells displayed markedly reduced expression of exhaustion markers (Figs. 1d, 4a, Figs. S1g, 6c).

To characterize the phenotypes of PB T cells enriched for tumor specificity, we identified all T cell clones that are present in both PB and tumor and contain at least one exhausted TIL (Fig. 4b). Interestingly, this analysis revealed that tumor-infiltrating Tex cells predominantly associate with PB Tem and Teff phenotypes. Of note, clonotype expansion may contribute to this observation (Fig. S6d). In contrast, PB *CD8*^+^ T cells clonally associated with non-exhausted TIL predominantly displayed naïve and central-memory phenotypes (Fig. 4c). Gene expression analyses comparing clonotype-matched TIL and PB T cells revealed that proliferation, effector, and exhaustion markers were upregulated within the TME, while markers expressed in memory T cells, such as *TCF7*, *GZMK*, or *KLRG1*, were downregulated in a subset of Tex cells (Fig. 4d).

To directly characterize activation-induced differentiation, we isolated *CD8*^+^ T cells from the peripheral blood of four separate, independent treatment-naïve HNSCC patients (Table S4). The corresponding *CD8*^+^ T cells were in vitro cultured in the absence or presence of TCR stimulation and subsequently subjected to scRNA/VDJ-seq (Fig. S6e, f). Consistent with the diversity of phenotypes identified in the clinical trial PB T cell analysis (Fig. 4a), we identified multiple T cell subsets, including Tem cells characterized by relatively high *GZMK* expression (Fig. S6g, i, k). Following TCR stimulation, different T cell phenotypes were less distinguishable, and cells displayed increased expression of proliferation and exhaustion markers (Fig. S6h, j, l).

We next focused on clonotypes that were shared between the unstimulated and stimulated conditions and associated with *GZMK*^+^ Tem cells in the unstimulated condition. Gene expression analysis revealed that TCR stimulation within individual Tem clonotypes leads to substantially reduced *GZMK* expression and a concomitant upregulation of proliferation, effector, and exhaustion genes (Fig. 4e, f, Fig. S6m). These results are consistent with gene expression changes that occur in TIL before and after NIT and suggest that TCR stimulation may be a main driver of Tem differentiation in vivo. Furthermore, although Tem clonotypes displayed a significant reduction in *GZMK* expression after TCR stimulation, greater levels of *GZMK* expression, compared to non-Tem clonotypes, were still detectable after four days of TCR stimulation. In summary, these data indicate that *GZMK*⁺ exhausted TILs likely originate from recently recruited and activated peripheral Tem cells undergoing differentiation in the TME.

### Pre-Tx GZMK-positivity within TILs predicts response to neoadjuvant immunotherapy

Activation and differentiation of tumor-specific T cells are crucial for effective anti-tumor immunity. To better understand how tumor-associated T cell differentiation relates to treatment response, we used *GZMK* expression as a marker of recent T cell activation and differentiation.

**Fig. 5:**
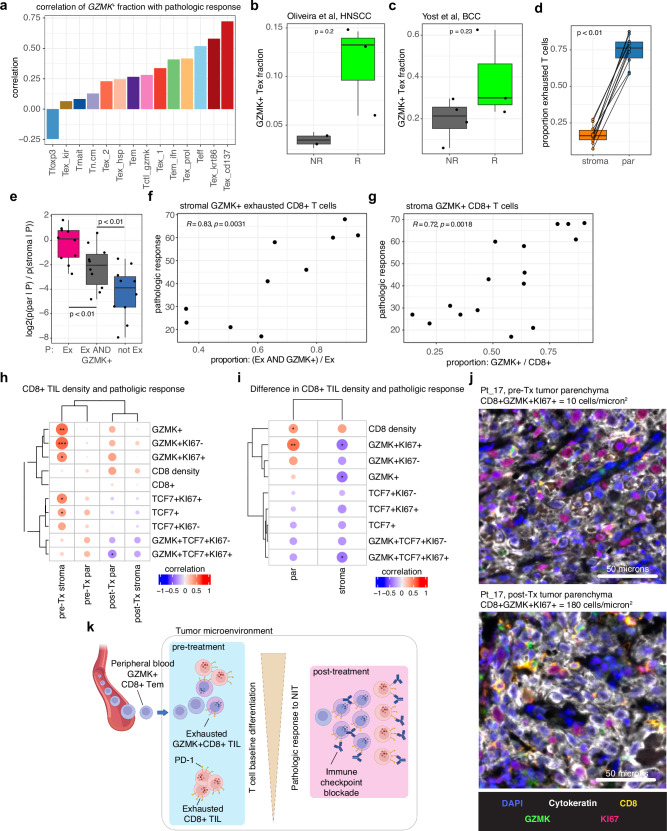
Pre-Tx GZMK transcript and protein expression are associated with pathological response. **a** Bar graph showing the correlation of *GZMK*-positivity within *CD8*^+^ TIL subsets and pathological response. Box plot showing GZMK-positivity within exhausted T cells for responders (R) to immunotherapy and non-responders (NR) isolated from pre-Tx biopsies of **b** basal cell carcinoma^71^ **c** or HNSCC^17^. *P*-value; Wilcoxon rank-sum test. **d** Box plot showing proportion of T cells that express exhaustion markers PD1 or TIM3 within the stroma or parenchyma (par) as measured by multispectral immunofluorescence. *P*-value; paired T test. **e** Box plot showing log2-transformed ratio of probabilities that T cells localize to either parenchymal (par) or stromal compartments conditioned on the T cell phenotype P. The following phenotypes were considered: the exhausted phenotype (Ex) comprises all T cells expressing PD1 and/or TIM3; Ex and GZMK^+^ T cells are characterized by the exhausted phenotype and GZMK expression; not exhausted refers to the complement of Ex. *P*-value; paired Wilcoxon rank-sum test. **f** Scatter plot showing the proportion of GZMK-positive and exhausted T cells compared to all exhausted T cells within the stroma and corresponding pathologic responses. Proportions are based on exhaustion panel multiplex IHC data. *P*-values are based on Fisher’s Z-transform. **g** Scatter plot showing the proportion of GZMK-positive T cells compared to all T cells within the stroma and corresponding pathologic responses. Proportions are based on progenitor panel multiplex IHC data. *P*-values are based on Fisher’s Z-transform. **h** Heatmap showing the correlation of indicated T cell populations, quantified as proportion of the total number of CD8+ T cells, with pathologic response for the indicated treatment states and anatomical compartments. Circle sizes are proportional to the absolute correlation. *P*-values are based on Fisher’s Z-transform; *: 0.05 ≥ *p*-value > 0.01; **: 0.01 ≥ *p*-value > 0.001; ***: 0.001 ≥ *p*-value > 0.0001. **i** Heatmap showing the correlation of the difference in proportion, obtained by subtracting the pre-Tx proportion from the post-Tx proportion, of the indicated T cell populations with pathologic response for the indicated treatment states and anatomical compartments. Circle sizes are proportional to the absolute correlation. *P*-values are based on Fisher’s Z-transform; *: 0.05 ≥ *p*-value > 0.01; **: 0.01 ≥ *p*-value > 0.001; ***: 0.001 ≥ *p*-value > 0.0001. **j** Micrographs showing representative multiplex immunofluorescence staining of pre- and post-Tx tumor sections of patient 17 displaying increased levels of parenchymal GZMK^+^KI67^+^ T cells following NIT. **k** Schematic summarizing the findings of this study. Created in BioRender (https://BioRender.com/ze97jrj).

Across a range of pre-Tx *CD8*^+^ TIL phenotypes, a higher proportion of *GZMK*⁺ cells was positively correlated with pathologic response following NIT (Fig. 5a, Table SI). A similar trend was also observed for *CD4*^+^ Tex subsets (Fig. S7a), and independent cohorts of HNSCC and basal cell carcinoma (Fig. 5b, c).

To validate these findings at the protein level and further study the anatomical localization of GZMK-positive TIL subsets, we generated multiplex immunofluorescence data using two different protein panels combining GZMK with either exhaustion or progenitor and proliferation markers in pre- and post-Tx clinical trial tumor specimens (Fig. 1a, Fig. S7b, Tables S2 and S5). Classification of stromal and parenchymal T cells revealed a substantial CD8^+^ T cell-enrichment within the tumor stroma (Fig. S7c). In addition, tumor-parenchymal CD8^+^ T cells were enriched in the exhausted phenotype compared to the stroma, likely indicating differences in antigen availability and an enrichment of tumor-specific T cells within the parenchyma (Fig. 5d). Notably, phenotype-based localization analysis revealed that GZMK^+^ Tex cells displayed an intermediate distribution between the stroma and parenchyma, when compared to GZMK^-^ Tex or non-exhausted subsets, possibly reflecting activation-induced upregulation of exhaustion markers in stromal GZMK^+^ T cells followed by parenchymal infiltration and gradual loss of GZMK expression (Fig. 5e).

Importantly, an elevated pre-Tx stromal proportion of *GZMK*^+^*CD8*^+^ Tex cells was associated with increased pathologic treatment response (Fig. 5f). Similarly, the overall stromal proportion of multiple *GZMK*^+^*CD8*^+^ and TCF7^+^ T subsets pre-Tx was also correlated with pathologic response (Fig. 5g, h, Fig. S7d). In contrast, pre-Tx parenchymal T cell levels were less predictive for treatment outcomes (Fig. 5h, Fig. S7e, f). These findings suggest that stromal T cell composition, particularly the enrichment of GZMK⁺ progenitor-like subsets, may reflect an enhanced baseline anti-tumor immunocompetence, associated with increased tumor-infiltration and differentiation of peripheral memory cells, underlying successful immunotherapy.

We also evaluated treatment-induced changes in proportions of different T cell subsets. Notably, both a post-Tx reduction of multiple stromal GZMK+ T cell populations and an increase of GZMK^+^KI67^+^ T cells within the tumor parenchyma were associated with increased pathological response (Fig. 5i), suggesting that increased parenchymal infiltration of GZMK^+^ T cell subsets may be associated with successful immunotherapy (Fig. 5i, j). Similar effects, albeit less pronounced, were observed in TCF7^+^CD8^+^ TIL subsets.

In summary, our findings suggest that high pre-treatment differentiation efficiency of GZMK^+^ progenitor-like T cells, likely recruited from the periphery, marks a state of enhanced immunocompetence associated with effective neoadjuvant immunotherapy (Fig. 5k).

### A pre-treatment *GZMK* gene expression signature predicts immunotherapy response across cancer types

**Fig. 6:**
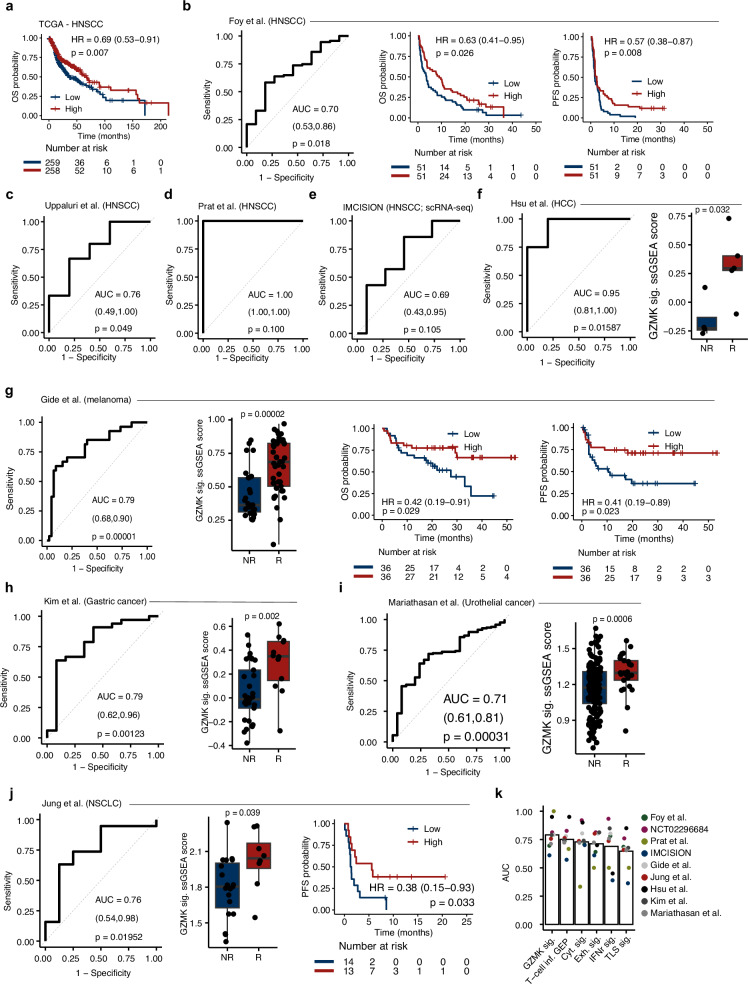
A pre-treatment GZMK gene expression signature predicts ICB response across multiple cancers in independent cohorts. **a** Kaplan–Meier overall survival (OS) curves comparing patients with low versus high *GZMK* signature scores in TCGA-HNSC (*n* = 518), stratified by the median. **b** Receiver operating characteristic (ROC) curve and corresponding area under the curve (AUC) value of the *GZMK* signature for predicting objective ICB response (left), OS (middle), and progression-free survival (PFS; right) in the cohort from Foy et al. (*n* = 102), stratified by median signature scores. ROC curves and AUC values of the *GZMK* signature for predicting objective ICB response in head and neck squamous cell carcinoma (HNSCC) cohorts: Uppaluri et al. (**c**; *n* = 20), Prat et al. (**d**; *n* = 5), and the IMCISION single-cell RNA-seq cohort (**e**; *n* = 18). For (**e**), pseudo-bulk gene expression of T cells was used to calculate the *GZMK* signature. **f** ROC curves, AUC values, and signature score distributions between responders and non-responders in Hsu et al. (**h**; *n* = 9). **g** ROC curve and AUC value of the *GZMK* signature for predicting objective ICB response (left), distribution of signature scores in responders versus non-responders (center), and Kaplan–Meier OS and PFS curves (right) stratified by median signature score in the cohort from Gide et al. (*n* = 72). **h**, **i** ROC curves, AUC values, and signature score distributions between responders and non-responders in additional ICB-treated cohorts: Kim et al. (**i**; *n* = 45), and Mariathasan et al. (**j**; *n* = 192). **j** ROC curve and AUC value for ICB response prediction (left), distribution of signature scores by response group (center), and Kaplan–Meier PFS curves (right) in the cohort from Jung et al. (*n* = 27). **k** Bar graph showing comparison of the predictive power of the *GZMK* gene signature for ICB response against established signatures across multiple clinical cohorts. Definitions of each established signature are provided in the “Methods” section. *P*-values in box plots in (**f**–**j**) were from a two-tailed Mann–Whitney U test. Hazard ratios (HRs) and *p*-values in (**a**, **b**, **g**, **j**) were from univariate Cox regression analysis. AUC values with 95% confidence intervals in (**b**–**j**) were estimated using bootstrapping, and *p*-values were computed using a Wilcoxon rank-sum test for difference from an AUC of 0.5.

To assess the generalizability of our single-cell observations, we derived a pre-treatment *GZMK* signature to predict patient outcomes undergoing standard-of-care or ICB treatment. Based on differential expression analyses of exhausted *GZMK*⁺ T cells, we curated a 40-gene signature (“Methods” section, Table S6) and first validated its prognostic relevance in the TCGA head and neck cancer cohort. Patients with high *GZMK* signature scores had significantly improved overall survival (OS; hazard ratio [HR] = 0.69, *P* = 0.007; Fig. 6a) following standard anti-cancer treatments.

We next tested the ability of the *GZMK* signature to predict ICB response across multiple independent HNSCC cohorts based on pre-treatment gene expression profiles. In the Foy et al. cohort^34^, pre-Tx signature expression scores were able to predict objective response (AUC = 0.70, *P* = 0.018), OS (HR = 0.63, *P* = 0.026), and progression-free survival following ICB (PFS; HR = 0.57, *P* = 0.008; Fig. 6b). Similarly, the *GZMK* signature predicted pathologic response in the Uppaluri et al.^3^ (AUC = 0.76, *P* = 0.049; Fig. 6c) and clinical response to ICB in the Prat et al.^35^ (AUC = 1.00, *P* = 0.10; Fig. 6d) cohorts. In the IMCISION trial^36^, where scRNA-seq data were available, the signature score calculated from T cell pseudo-bulk expression also predicted pathologic response to ICB (AUC = 0.69, *P* = 0.105; Fig. 6e), suggesting its applicability across platforms.

Extending our analysis beyond HNSCC, we found that the pre-treatment *GZMK* signature retained predictive value for ICB response across diverse tumor types. In multiple independent ICB-treated cohorts of melanoma^37^, non-small cell lung cancer (NSCLC)^38^, hepatocellular carcinoma (HCC)^39^, gastric cancer^40^, and urothelial cancer^41^, high pre-treatment signature scores were consistently associated with objective response and, when available, longer OS and/or PFS (AUCs ranging from 0.71 to 0.95; Fig. 6f–j). In all datasets, responders exhibited significantly higher *GZMK* signature scores than non-responders (Fig. 6f–j). To contextualize the performance of the *GZMK* signature, we benchmarked it against several established ICB response signatures, including cytotoxic T cell^42^, exhausted T cell^43^, interferon-γ^44^, tertiary lymphoid structure (TLS)^45^, and the T cell–inflamed gene expression profile (GEP)^44^ signatures. Notably, the *GZMK* signature demonstrated the highest average predictive accuracy across all cohorts, with an average AUC of 0.79 (Fig. 6k).

Collectively, these findings demonstrate that a pre-treatment *GZMK* gene expression signature, possibly reflecting T cell-intrinsic activation programs, has strong and consistent predictive value for response to ICB therapy across multiple cancer types. This highlights a central role of GZMK⁺ T cell populations in effective anti-tumor immunity and establishes a clinically actionable biomarker candidate for patient stratification.

## Discussion

In this study, we describe developmental hierarchies and phenotypic compartmentalization associated with tumor-infiltrating T cells isolated from patients with newly diagnosed advanced HNSCC in the context of NIT. Single-cell transcriptomic and TCR-based clonal analysis revealed a substantial phenotypic diversity within both *CD4*^+^ and *CD8*^+^ T cell compartments. In treatment-naïve tumors, T cell clones exhibited phenotypic compartmentalization; *CD4*^+^ T cells largely separated into Treg and non-Treg phenotypes, including Tex phenotypes, suggesting limited developmental plasticity within the respective T cell clones, consistent with previous observations^14^. Similarly, *CD8*^+^ T cell clones clustered around three central phenotypes: exhausted, tissue-resident memory-like cells (Tex_krt86), central memory/naïve (Tn.cm), and effector-memory (Tem) cells. Tex_krt86-associated phenotypes were associated with reduced pre-Tx peripheral frequencies, consistent with a tissue-resident phenotype. In contrast, phenotypic clusters associated with central and effector-memory phenotypes displayed substantially greater frequencies in circulation, suggesting that different T cell clones may experience a degree of phenotypic and physical segregation prior to treatment. The presence of segregated T cell phenotypes across T cell clones may reflect reduced differentiation of tumor-reactive T cells and indicate an increased isolation of the TME from extra-tumoral sites involved in anti-tumor immunity underlying impaired anti-cancer immunity in the untreated state^8,9,46^.

In contrast, the increased phenotypic diversity within Tex_krt86-associated T cell clones post-Tx suggests enhanced treatment-induced differentiation of *GZMK*-expressing progenitor T cells within the TME. Additionally, analysis of *CD8*^+^ T cells isolated from patient-derived blood samples suggests that *GZMK* expression within the exhausted T cell compartment can serve as a marker of recent tumor-infiltration and differentiation. Consistently, a so-called ‘progenitor exhausted’ T cell population that is also characterized by GZMK expression has been described in lung cancer and shown to accumulate in post-treatment samples of responders to immunotherapy^47,48^. Similarly, in hepatocellular carcinoma, *GZMK*⁺ *CD8*⁺ effector/effector-memory T cells have been identified as critical mediators of response to immunotherapy^49^.

Our analysis further shows that higher baseline differentiation of *GZMK*+ progenitor T cells within treatment-naïve tumors is associated with increased pathologic response. The increased frequency of differentiated *GZMK*^+^ TIL within the TME may reflect enhanced recruitment and activation of peripheral progenitor T cells that have elevated potential to respond to NIT. Increased pathologic responses have been associated with improved recurrence-free survival in different cancers^50–52^. Therefore, GZMK-based assessment of pre-Tx T cell differentiation dynamics may offer a path toward more personalized NIT strategies. We also observed a significant association of pre-treatment TCF7 expression and pathologic response, consistent with findings made in melanoma^18^.

Moreover, we extended the clinical relevance of our findings to other cancer indications by developing a transcriptional signature derived from GZMK⁺ T cell subsets, which was able to predict response and survival following ICB across diverse cancer types and independent cohorts using pre-treatment gene expression profiles only. This suggests a generalizable role for *GZMK*⁺ T cells as indicators of pre-existing anti-tumor immunocompetence.

We also show that a subset of Tex TILs, characterized by the expression of tissue residency markers (*ITGAE*/CD103), are found at greater frequency in the post-Tx peripheral blood, suggesting egress from the tumor into circulation following NIT, consistent with observations we made previously^14^. Recent data indicating that relapse of distant metastatic disease is substantially reduced in patients receiving neoadjuvant immune checkpoint blockade, suggesting enhancement of systemic anti-tumor immunity^5^, may point to the clinical relevance of this observation. In addition, a subset of Tem-associated clonotypes, characterized by relatively high frequencies in circulation pre- and post-Tx, displayed significantly increased signs of differentiation in the tumor post-Tx, consistent with reduced activation or tumor-parenchymal infiltration of pre-Tx T cells. These findings also suggest that the emergence of exhausted T cells within individual TIL clonotypes post-Tx that did not harbor exhausted T cells pre-Tx may also contribute to greater detection frequencies of exhausted T cells in the periphery after NIT. Consistent with this idea, TCR stimulation of peripherally derived Tem-like cells in vitro led to the upregulation of proliferation, effector, and exhaustion markers and a concomitant downregulation of GZMK. However, how therapeutics, such as anti-PD-L1 and TGF-β blockade, may affect such developmental processes in vivo requires additional study. Furthermore, the origin of peripheral Tem cells with respect to distinct anatomical sites, such as tumors or tumor-draining lymph nodes, requires additional analysis. It is possible that *GZMK*^+^ Tem cells represent an intermediate cell state that originates from the lymph nodes, traffics into the tumor via peripheral circulation, to undergo additional differentiation upon antigen exposure. Further analysis of the factors underlying the differences in T cell recruitment and differentiation is also warranted.

GZMK has recently been associated with complement activation^53,54^. If GZMK-mediated complement activation also contributes to improved response to immunotherapy, as proposed for other lymphocyte populations^55^, requires further evaluation.

Limitations to this analysis exist. Clonotype classification may be confounded by false negatives. Tumor specificity of T cells has been largely inferred based on transcriptional phenotypes. scRNA/VDJ-seq is limited in cell number and does not capture the entire complexity of the TCR repertoire. The use of bispecific bintrafusp alfa prevents unambiguous attribution of observed treatment effects to either PD-L1 blockade or TGF-β inhibition, which may, for instance, both affect downstream TCR signaling^56^. The analyses linking the *GZMK* signature to ICB response were based on retrospective cohorts, and thus subject to patient selection bias and treatment heterogeneity; however, we mitigated this by validating our findings across multiple independent, multi-center datasets. While GZMK⁺ TILs emerged as a compelling biomarker of immunocompetence, the cellular ontogeny, trafficking patterns, antigen specificity, and direct functional roles of these cells remain incompletely understood and warrant dedicated mechanistic investigation in future studies. Further protein-level validation using multiparameter flow cytometry was limited by the lack of additional clinical specimens and the availability of a compatible and internally validated anti-human granzyme K antibody.

In conclusion, our study illustrates phenotypic plasticity and differentiation potential of *GZMK*+ progenitor T cells within tumors and highlights the predictive and functional relevance of GZMK⁺ progenitor T cells for immunotherapy outcomes. Furthermore, these findings provide insights into the mechanisms underlying the reinvigoration of anti-cancer immunity and suggest a path toward biomarker-driven personalization of immunotherapy.

## Methods

### Patient samples

Pre- and post-NIT treatment blood and tumor specimens were collected under a National Institutes of Health (NIH) IRB-approved (approval number 20-C-0024) clinical trial (NCT04247282, clinicaltrials.gov, registered 01/30/2020) following informed consent as reported previously^4,20^. Blood specimens from additional patients with advanced cancer were collected under an NIH IRB-approved (approval number 18-DC-0051) biospecimen protocol (NCT03429036, clinicaltrials.gov, registered 11/06/2020) following informed consent. All research was conducted in accordance with the Declaration of Helsinki.

### Pathologic responses

Digital pathology software (QuPath) assessment of H&E slides from each surgical specimen was used to quantify the pathologic tumor response (pTR) in the primary tumor, defined as the area percentage of viable tumor within the tumor bed of the primary tumor and involved cervical lymph nodes (surface area of residual viable tumor/surface area of total tumor bed x 100)^57,58^.

### Single-cell RNA/VDJ-sequencing of tumor and peripheral T cells

Fresh pre- and post-treatment tumor biopsies were digested using the Human Tumor Dissociation Kit and the gentleMACS Dissociator (Miltenyi Biotec) per manufacturer recommendations. Single-cell tumor suspensions were washed with RPMI-1640, passed through a 70-μm filter, stained with TruStain FcX (Biolegend), a primary-conjugated anti-human CD3 antibody (clone SK7, Biolegend), and the viability marker Sytox Blue (Thermo), and viable CD3-positive cells were isolated using fluorescent activated cell sorting (FACSAria III, BD Biosciences). Cryopreserved post-treatment PBMC were thawed, and T cells were isolated using the EasySep Human T Cell Isolation Kit (Stemcell) per manufacturer recommendations. Cells were washed in 1× PBS, filtered (70 μm), and quantified using acridine orange/propidium iodide (AO/PI) staining on a Cellometer Auto 2000 (Nexcelom). Cells were concentrated to 1000 cells/μL and loaded onto the Chromium Controller (10X Genomics) with a target of 6000 cells per sample. Cells were mixed with barcoded gelbeads and 5′ GEM Kit v1.1 or v2 reagents (10X Genomics), and single-cell capture was performed. Following reverse transcription, cDNA was amplified, and gene expression and TCR sequencing libraries were constructed according to the manufacturer’s recommendations. Each DNA library was loaded into a sequencing lane on a NovaSeq or NextSeq system (Illumina) and was sequenced with pair-end reads of 75 bp.

### scRNA/VDJ-seq analysis

Base calling and de-multiplexing, allowing one mismatch, were performed using RTA (v3.4.4) and Bcl2fastq (v2.20), respectively. The resulting fastq files were processed using the 10x Genomics Cell Ranger software (v6.1.2) using default parameters and reference genomes hg38-2020-A and GRCh88-5.0.0. TCR clonotypes, shared between different samples from the same patient, were identified using Cell Ranger Aggr with default parameters. Downstream analysis was performed using the P programming language^59^ and the R package Seurat^60^ on the merged data. The scRNA/VDJ-seq datasets from TIL, matched post-Tx peripheral-blood T cells, and in vitro TCR-stimulated T cells were all analyzed separately to minimize the impact of batch effects. Doublets were identified using the R package scds^61^ and scds::cxds_bcds_hybrid, assuming a doublet rate of 0.039, and removed. Only cells satisfying the following conditions were kept for the analysis: nFeature_RNA ≥ 250 & nFeature_RNA ≤ 5000 & nCount_RNA ≥ 500 & percent.mt ≤ 25 & HBA_HBB ≤ 25 using Seurat nomenclature. Module scores of gene sets were obtained using Seurat::AddModuleScore with default parameters. CD4+ and CD8+ T cells were identified based on clonotype-level CD4 and CD8A/B expression, respectively. Single-cell data was normalized using Seurat::NormalizeData. Variable features were identified using Seurat::FindVariableFeatures(assay = ‘RNA’, selection.method = ‘vst’, nfeatures = 2500); TCR and IGH/L genes were removed from the variable features, and the data was scaled using Seurat:: ScaleData(vars.to.regress = ‘nCount_RNA’). PCA was performed using Seurat::RunPCA. Data was harmonized^62^ at the patient level using Seurat::RunHarmony(). Graph-based clustering was performed using Seurat::FindNeighbors and Seurat::FindClusters using resolutions 1.2 and 0.6 for CD4^+^ - and CD8^+^ T cells, respectively. UMAPs were obtained using Seurat::RunUMAP. Proliferating CD4^+^ T cells were subclustered to resolve Treg and non-Treg subsets. Proliferating CD8^+^ Tex cells were further subdivided into GZMK^+^ or GZMK^-^ subsets based on detectable GZMK expression. Differential gene expression analysis between different T cell subsets was performed using Seurat::FindAllMarkers. Gene set enrichment analysis was performed using clusterProfiler::compareCluster()^63^, considering GO, KEGG, or Reactome terms. Additional data processing and visualization were performed using tidyverse^64^, ggplot2^65^, and ComplexHeatmaps^66^. Phenotype associations, i.e., the probabilities of observing two phenotypes with the same T cell clone, were estimated using relative frequencies for each clonotype. The resulting estimates were subjected to weighted averaging using normalized, log10-transformed clone sizes.

Hierarchical clustering was based on Euclidean distances. Box plots represent the following information: horizontal lines inside each box indicate the median, the top and bottom of the box correspond to the interquartile range, and vertical bars indicate the 5th and 95th percentiles.

### Peripheral blood and tumor TCRβ-sequencing

Total peripheral-blood T cells were isolated from cryopreserved pre- and post-treatment PBMC using the EasySep Human T cell Isolation Kit (Stemcell Technologies) per manufacturer recommendations. Genomic DNA was extracted from peripheral-blood T cells or pre-treatment tumor using the DNA Mini Kit (Qiagen) per manufacturer's recommendations. Deep TCRβ sequencing was performed using the Milaboratories gDNA human TCRα/β kit and MiixCR software per manufacturer recommendations by the Center for Cancer Research Genomics Core. Peripheral or tumor TCRβ sequences were associated with scRNA-sequenced T cells using the CDR3 nucleic acid sequence, allowing zero mismatches.

### Ex vivo stimulation of peripheral-blood CD8+ T cells

CD8+ peripheral-blood T cells were isolated from fresh PBMC using the EasySep Human CD8+ T cell Isolation Kit (Stemcell Technologies) per manufacturer recommendations. 3 × 10^5^ cells suspended in PRMI-1640-based media supplemented with 10% fetal bovine serum (FBS), 5 mM glutamine, and 25 mM 4-(2-hydroxyethyl)-1-piperazineethanesulfonic acid (HEPES) were plated in a 12-well tissue culture plate and cultured for 4 days in the presence or absence of Human T-Activator CD3/28 Dynabeads (Thermo Fisher) used per manufacturer recommendations. After culture, cells were harvested, magnetic stimulation beads were removed, and cells were subjected to capture for single-cell RNA- and TCR-sequencing.

### Multispectral immunofluorescence

Formalin-fixed paraffin-embedded tumors were sectioned at 5 μm, baked at 60 °C for 30 min, soaked in Bond Dewax Solution (Leica), and rehydrated. Deparaffinization and staining of all slides were performed on the Leica BOND RX Autostainer (Leica). Before being used in combination, the specificity and optimal dilution of each antibody were individually determined with chromogenic immunohistochemistry (3′-3′ diaminobenzidine tetrahydrochloride hydrate; DAB) using slides from normal tonsil and head and neck carcinoma, consistent with best-practice guidelines^67^. Heat-induced epitope retrieval was performed by heating to 95 °C in BOND epitope retrieval solutions ER1 or ER2 (Leica). Tyramine Signal Amplification (TSA) Opal technology was used for immunofluorescence staining. After individual primary antibody optimization, primary and secondary antibody and opal pairings were optimized for minimum background and desired signal amplification in monoplex immunofluorescence using head and neck carcinoma sections. Antibody and amplification reagents are listed in Table S5. Slides were coverslipped using the Leica CV5030 automated coverslipper after staining. Whole slide images were obtained at 40× magnification using 7-color whole slide unmixing filters on a Vectra Polaris. All paired pre- and post-treatment tumors were stained and scanned concurrently.

### Immunofluorescence analysis

Whole slide analysis of each stained slide was performed with HALO Image Analysis software (v3.3, Indica Labs). Whole slide annotations were performed using the Random Forest Tissue Classifier Algorithm. Standard nuclear segmentation was used. The HALO AI Nuclear Segmentation Classifier was trained to identify nuclei of various sizes and used for the CD8-positive cell detection. Fluorescence intensities of each marker for each cell were determined using the HALO Highplex FL Analysis Algorithm. Separate Highplex FL Analysis Algorithms were used for tumor and immune cells, given differences in nuclear size. Common fluorescence thresholds used to assign positivity or scaled intensity (1+, 2+, or 3+) for a given marker were used for each patient’s paired pre- and post-treatment tumor to determine PD-L1 expression. CD8-positive cell density was defined as the absolute number of positive cells per unit area (mm^2^). H-score was used to describe protein expression on cells and was defined as: (% of 1+ cells × 1)+(% of 2+ cells × 2)+(% of 3+ cells × 3) for a range of 0–300.

### The GZMK gene expression signature and its clinical relevance analysis

The *GZMK* signature was derived through differential expression analysis of pre-treatment tumor-infiltrating *CD8*⁺ T cells using the Seurat package (v4.0.0) in R. Specifically, we compared *GZMK*-expressing Tex_krt86 and Tex_cd137 cells with *GZMK*^-^ *CD8*⁺ T cells of all other CD8^+^ T cell phenotypes using Seurat::FindMarkers() with default parameters. A total of 40 genes that were significantly upregulated in the target populations were selected to construct the *GZMK* signature gene set (Table S6). Gene signature scoring was performed using single-sample gene set enrichment analysis (ssGSEA) using the R package GSVA^68^. For bulk RNA-seq datasets, the *GZMK* signature score was computed directly from normalized bulk expression data. For scRNA-seq datasets, the signature was computed using pseudo-bulk expression derived from T cells within each sample. Only pre-treatment samples were included in the clinical correlation analyses across all external public datasets used for assessing the association between the *GZMK* signature and patient outcomes.

To benchmark the predictive performance of the GZMK signature, we compared it against several established immune-related gene expression signatures previously associated with ICB response: Cytotoxic T cell signature (Cyt): GZMA, PRF1^42^; Exhausted T cell signature (Exh): PDCD1, CTLA4, LAG3, HAVCR2, TIGIT^43^; Interferon-γ signature (IFNγ): IDO1, CXCL10, CXCL9, HLA-DRA, STAT1, IFNG^44^; Tertiary lymphoid structure (TLS) signature: BCL6, CD86, CXCR4, LAMP3, SELL, CCR7, CXCL13, CCL21, CCL19^45^; T cell–inflamed gene expression profile (T cell–inf GEP): CD276, HLA-DQA1, CD274, IDO1, HLA-DRB1, HLA-E, CMKLR1, PDCD1LG2, PSMB10, LAG3, CXCL9, STAT1, CD8A, CCL5, NKG7, TIGIT, CD27, CXCR6^44^. Comparisons of *GZMK* signature scores between two groups were performed using the two-tailed Mann–Whitney U test. Receiver operating characteristic (ROC) curves and area under the curve (AUC) values with 95% confidence intervals were calculated using the ci.auc() function from the *pROC* package^69^, which applies stratified bootstrapping with 2000 replicates. *P*-values for AUC estimates were computed using the roc.area() function from the *verification* package, which implements a Wilcoxon rank-sum test to assess deviation from an AUC of 0.5.

Survival analyses were conducted using the R packages *survminer* (v0.4.9) and *survival* (v3.3.1). Hazard ratios (HRs) with 95% confidence intervals and corresponding *p*-values were estimated using univariate Cox proportional hazards regression with the coxph() function, as implemented in the *survival* package [Therneau, T.M. & Grambsch, P.M. Modeling survival data: Extending the Cox model, (Springer-Verlag, New York, 2000); Therneau, T. A package for survival analysis in S. R package version 2(2015).].

### Statistics

All statistical analyses were conducted using R (v4.3.0). *P*-values were computed using Wilcoxon rank-sum or T-tests as indicated. Paired tests were performed when applicable. *P*-values associated with Pearson correlations were based on Fisher’s Z-transform. All statistical tests were two-sided unless otherwise specified.

## Supplementary information

chcklist

Supplementary information

## Data Availability

All raw and processed sequencing data generated in this study are publicly available through the Gene Expression Omnibus (GEO). This includes single-cell RNA-seq data of tumor-infiltrating T cells from pre- and post-treatment tumors (GSE296954), matched post-Tx peripheral-blood T cells (GSE296867), and peripheral-blood CD8+ T cells from four separate donors used for in vitro stimulation experiments (GSE296771). Additional processed data and peripheral-blood deep TCRβ sequencing data are available at https://gitlab.com/sop_public/gzmk-hnscc. Previously published datasets used in this study are publicly accessible. For the TCGA-HNSC cohort, bulk RNA-seq data were downloaded from UCSC Xena (https://xena.ucsc.edu/) with TPM normalization applied^70^, and corresponding clinical annotations were obtained from the Genome Data Commons (https://portal.gdc.cancer.gov/). For the immunotherapy response analyses, data from the Foy et al. cohort^34^ were accessed via GEO (GSE159067); data from the Prat et al. HNSCC cohort^35^ were obtained from GEO (GSE93157), and data from the Uppaluri et al.^3^ were provided directly by the original authors. For the IMCISION trial^36^, single-cell RNA-seq data were downloaded from GEO (GSE232240). Additionally, ICB-treated cohorts included the Gide et al. dataset^37^, retrieved from the European Nucleotide Archive (PRJEB23709); the Jung et al. dataset^38^, available from GEO (GSE135222); the Hsu et al. dataset^39^, available from GEO (GSE140901); the Kim et al. dataset^40^, obtained from the European Nucleotide Archive (PRJEB25780); and the Mariathasan et al. dataset^41^, available via the European Genome-Phenome Archive (EGAS00001002556).
