# Supplementary material for: GZMK expression within activated intratumoral T-cell subsets reflects differentiation efficiency and predicts response to cancer immunotherapy
Source: NPJ Precis Oncol. 2026 Apr 18;10:237. doi: 10.1038/s41698-026-01437-7 (PMC13280249; doi:10.1038/s41698-026-01437-7)
Supplement: Supplementary file 1 — chcklist [file 41698_2026_1437_MOESM1_ESM.pdf]

Supplemental figures, tables and data for:

GZMK expression within activated intratumoral T-cell subsets reflects differentiation efficiency and predicts response to cancer immunotherapy

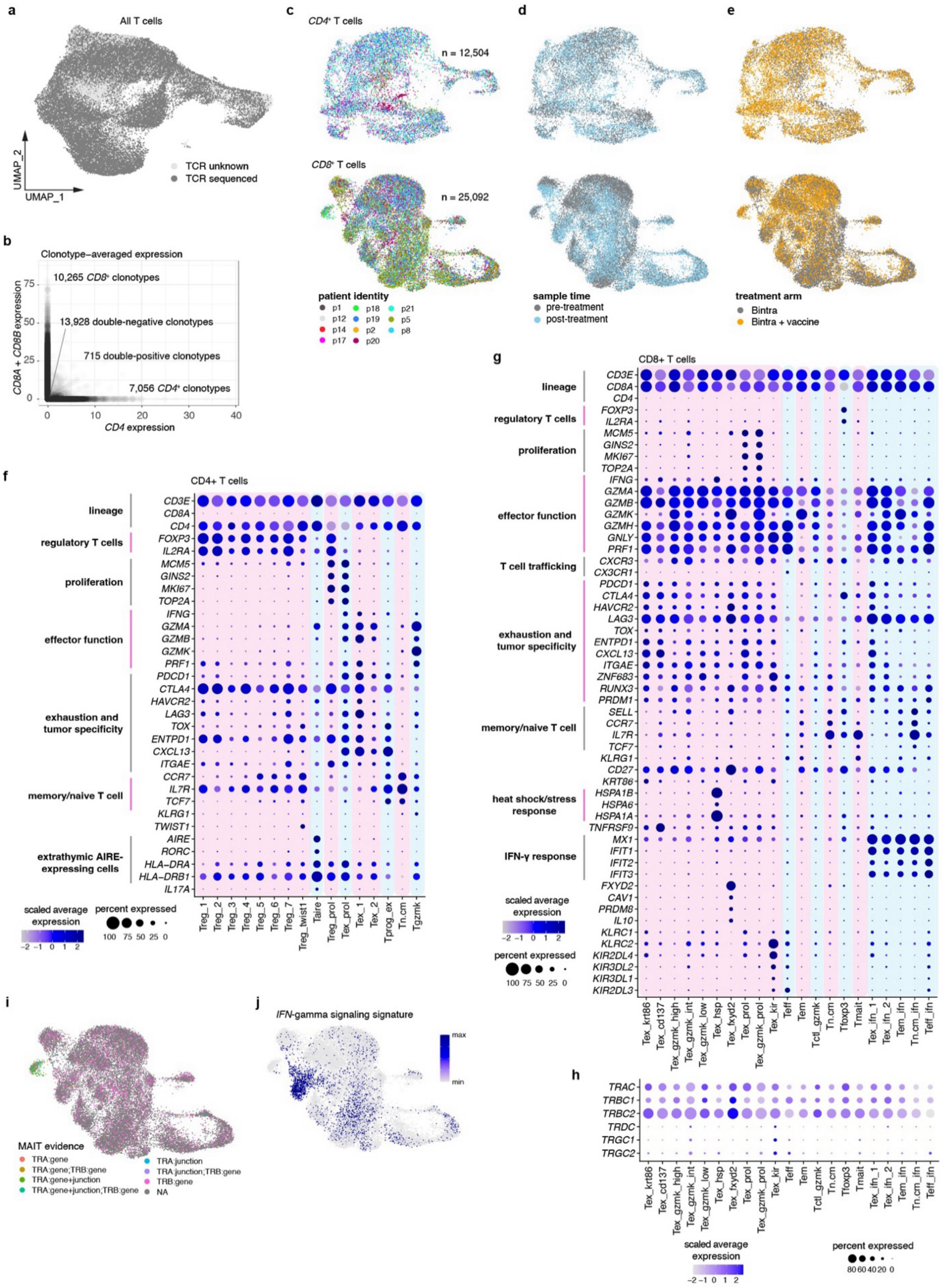

a
All T cells
UMAP_2
UMAP_1
TCR unknown
TCR sequenced
b
Clonotype–averaged expression
10,265 CD8+ clonotypes
13,928 double-negative clonotypes
715 double-positive clonotypes
7,056 CD4+ clonotypes
CD8A + CD8B expression
CD4 expression
c
CD4+ T cells
n = 12,504
CD8+ T cells
n = 25,092
patient identity
p1 p12 p14 p17 p18 p19 p2 p20 p21 p5 p8
d
sample time
pre-treatment
post-treatment
e
treatment arm
Bintra
Bintra + vaccine
f
CD4+ T cells
lineage
regulatory T cells
proliferation
effector function
exhaustion and tumor specificity
memory/naive T cell
extrathymic AIRE-expressing cells
CD3E CD8A CD4 FOXP3 IL2RA MCM5 GINS2 MKI67 TOP2A IFNG GZMA GZMB GZMK PRF1 PDCD1 CTLA4 HAVCR2 LAG3 TOX ENTPD1 CXCL13 ITGAE CCR7 IL7R TCF7 KLRG1 TWIST1 AIRE RORC HLA–DRA HLA–DRB1 IL17A
Treg_1 Treg_2 Treg_3 Treg_4 Treg_5 Treg_6 Treg_7 Treg_twist1 Taire Treg_prol Tex_prol Tex_1 Tex_2 Tprog_ex Tn.cm Tgzmk
scaled average expression
percent expressed
g
CD8+ T cells
lineage
regulatory T cells
proliferation
effector function
T cell trafficking
exhaustion and tumor specificity
memory/naive T cell
heat shock/stress response
IFN-γ response
CD3E CD8A CD4 FOXP3 IL2RA MCM5 GINS2 MKI67 TOP2A IFNG GZMA GZMB GZMK GZMH GNLY PRF1 CXCR3 CX3CR1 PDCD1 CTLA4 HAVCR2 LAG3 TOX ENTPD1 CXCL13 ITGAE ZNF683 RUNX3 PRDM1 SELL CCR7 IL7R TCF7 KLRG1 CD27 KRT86 HSPA1B HSPA6 HSPA1A TNFRSF9 MX1 IFIT1 IFIT2 IFIT3 FXYD2 CAV1 PRDM8 IL10 KLRC1 KLRC2 KIR2DL4 KIR3DL2 KIR3DL1 KIR2DL3
Tex_krt86 Tex_cd137 Tex_gzmk_high Tex_gzmk_int Tex_gzmk_low Tex_hsp Tex_fxyd2 Tex_prol Tex_gzmk_prol Tex_kir Teff Tem Tctl_gzmk Tn.cm Tfoxp3 Tmait Tex_ifn_1 Tex_ifn_2 Tem_ifn Tn.cm_ifn Teff_ifn
scaled average expression
percent expressed
h
TRAC TRBC1 TRBC2 TRDC TRGC1 TRGC2
scaled average expression
percent expressed
i
MAIT evidence
TRA:gene
TRA:gene;TRB:gene
TRA:gene+junction
TRA:gene+junction;TRB:gene
TRA:junction
TRA:junction;TRB:gene
TRB:gene
NA
j
IFN-gamma signaling signature
max
min

Figure S1. Cell-type classification of tumor-infiltrating T cell.

a, Scatter plot showing UMAP embedding all tumor-infiltrating T cells colored availability of TCR sequence.

b, Scatter plot showing clonotype-based expression averages of *CD4* and *CD8* (*CD8A* + *CD8B*) used to identify $CD4^+$ and $CD8^+$ T cells.

c, d, e, Scatter plot showing UMAP embeddings of $CD4^+$ (top) and $CD8^+$ (bottom) T cells colored by (c) patient identity, (d) sample time relative to treatment or (e) treatment arm.

f, g, h, Dot plots showing scaled average expression of T cell marker genes in (f) $CD4^+$ and (g,h) $CD8^+$ T cells within indicated T cell subsets. Circle size corresponds to fraction of cells with detectable expression for a given gene. Alternating background colors group similar T cell subsets.

i, Scatter plot showing UMAP embedding of $CD8^+$ T cells. Colors indicate presence TCR gene segments associated with mucosal-associated invariant T cells (MAITs).

j, Scatter plot showing UMAP embedding of $CD8^+$ T cells colored by IFN-γ signature expression.

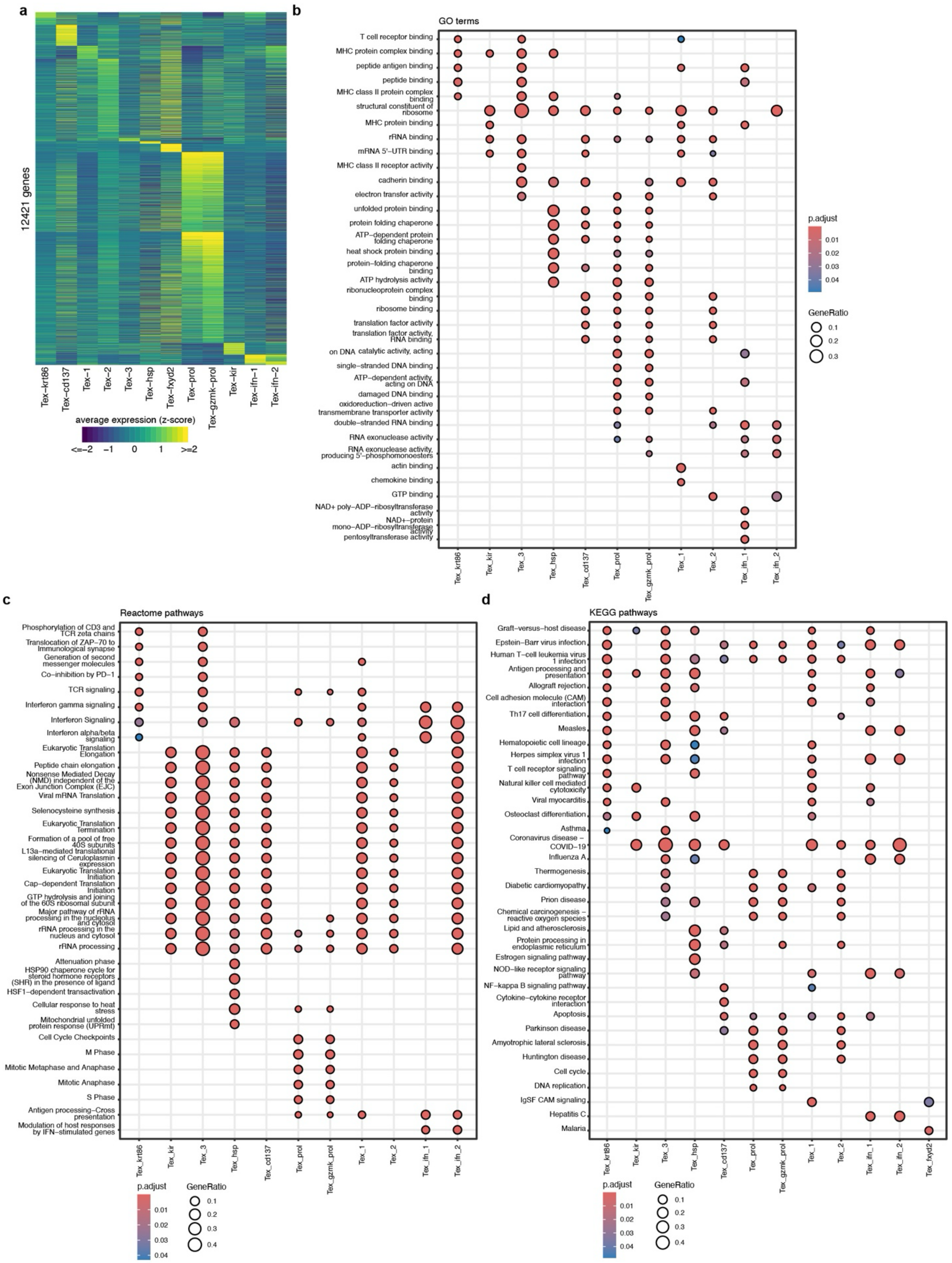
a
12421 genes
Tex–krt86
Tex–cd137
Tex–1
Tex–2
Tex–3
Tex–hsp
Tex–fxyd2
Tex–prol
Tex–gzmk–prol
Tex–kir
Tex–ifn–1
Tex–ifn–2
average expression (z-score)
<=–2 –1 0 1 >=2
b
GO terms
T cell receptor binding
MHC protein complex binding
peptide antigen binding
peptide binding
MHC class II protein complex binding
structural constituent of ribosome
MHC protein binding
rRNA binding
mRNA 5'–UTR binding
MHC class II receptor activity
cadherin binding
electron transfer activity
unfolded protein binding
protein folding chaperone
ATP–dependent protein folding chaperone
heat shock protein binding
protein–folding chaperone binding
ATP hydrolysis activity
ribonucleoprotein complex binding
ribosome binding
translation factor activity
translation factor activity, RNA binding
catalytic activity, acting on DNA
single–stranded DNA binding
ATP–dependent activity, acting on DNA
damaged DNA binding
oxidoreduction–driven active transmembrane transporter activity
double–stranded RNA binding
RNA exonuclease activity
RNA exonuclease activity, producing 5'–phosphomonoesters
actin binding
chemokine binding
GTP binding
NAD+ poly–ADP–ribosyltransferase activity
NAD+–protein mono–ADP–ribosyltransferase activity
pentosyltransferase activity
Tex_krt86
Tex_kir
Tex_3
Tex_hsp
Tex_cd137
Tex_prol
Tex_gzmk_prol
Tex_1
Tex_2
Tex_ifn_1
Tex_ifn_2
p.adjust
0.01
0.02
0.03
0.04
GeneRatio
0.1
0.2
0.3
c
Reactome pathways
Phosphorylation of CD3 and TCR zeta chains
Translocation of ZAP–70 to Immunological synapse
Generation of second messenger molecules
Co–inhibition by PD–1
TCR signaling
Interferon gamma signaling
Interferon Signaling
Interferon alpha/beta signaling
Eukaryotic Translation Elongation
Peptide chain elongation
Nonsense Mediated Decay (NMD) independent of the Exon Junction Complex (EJC)
Viral mRNA Translation
Selenocysteine synthesis
Eukaryotic Translation Termination
Formation of a pool of free 40S subunits
L13a–mediated translational silencing of Ceruloplasmin expression
Eukaryotic Translation Initiation
Cap–dependent Translation Initiation
GTP hydrolysis and joining of the 60S ribosomal subunit
Major pathway of rRNA processing in the nucleolus and cytosol
rRNA processing in the nucleus and cytosol
rRNA processing
Attenuation phase
HSP90 chaperone cycle for steroid hormone receptors (SHR) in the presence of ligand
HSF1–dependent transactivation
Cellular response to heat stress
Mitochondrial unfolded protein response (UPRmt)
Cell Cycle Checkpoints
M Phase
Mitotic Metaphase and Anaphase
Mitotic Anaphase
S Phase
Antigen processing–Cross presentation
Modulation of host responses by IFN–stimulated genes
Tex_krt86
Tex_kir
Tex_3
Tex_hsp
Tex_cd137
Tex_prol
Tex_gzmk_prol
Tex_1
Tex_2
Tex_ifn_1
Tex_ifn_2
p.adjust
0.01
0.02
0.03
0.04
GeneRatio
0.1
0.2
0.3
0.4
d
KEGG pathways
Graft–versus–host disease
Epstein–Barr virus infection
Human T–cell leukemia virus 1 infection
Antigen processing and presentation
Allograft rejection
Cell adhesion molecule (CAM) interaction
Th17 cell differentiation
Measles
Hematopoietic cell lineage
Herpes simplex virus 1 infection
T cell receptor signaling pathway
Natural killer cell mediated cytotoxicity
Viral myocarditis
Osteoclast differentiation
Asthma
Coronavirus disease – COVID–19
Influenza A
Thermogenesis
Diabetic cardiomyopathy
Prion disease
Chemical carcinogenesis – reactive oxygen species
Lipid and atherosclerosis
Protein processing in endoplasmic reticulum
Estrogen signaling pathway
NOD–like receptor signaling pathway
NF–kappa B signaling pathway
Cytokine–cytokine receptor interaction
Apoptosis
Parkinson disease
Amyotrophic lateral sclerosis
Huntington disease
Cell cycle
DNA replication
IgSF CAM signaling
Hepatitis C
Malaria
Tex_krt86
Tex_kir
Tex_3
Tex_hsp
Tex_cd137
Tex_prol
Tex_gzmk_prol
Tex_1
Tex_2
Tex_ifn_1
Tex_ifn_2
Tex_fxyd2
p.adjust
0.01
0.02
0.03
0.04
GeneRatio
0.1
0.2
0.3
0.4

Figure S2. Gene expression differences across CD8$^+$ Tex subsets.

a, Heatmap showing row standardized average gene expression of genes upregulated in indicated CD8 Tex clusters (Supplemental Table II). Total number significantly upregulated genes per cluster (adjuststed p-value <= 0.05): Tex_krt86: 454; Tex_cd137: 738; Tex_1: 460; Tex_2: 2776; Tex_3: 124; Tex_hsp: 86; Tex_fxyd2: 290; Tex_prol: 2813; Tex_gzmk_prol: 3916; Tex_kir 406; Tex_ifn_1: 241; Tex_ifn_2: 117.

b, c, d, dot plot showing enrichment of GO terms (b), Reactome pathways (c) and KEGG pathways (d) within genes upregulated in specific Tex clusters shown in (**a**).

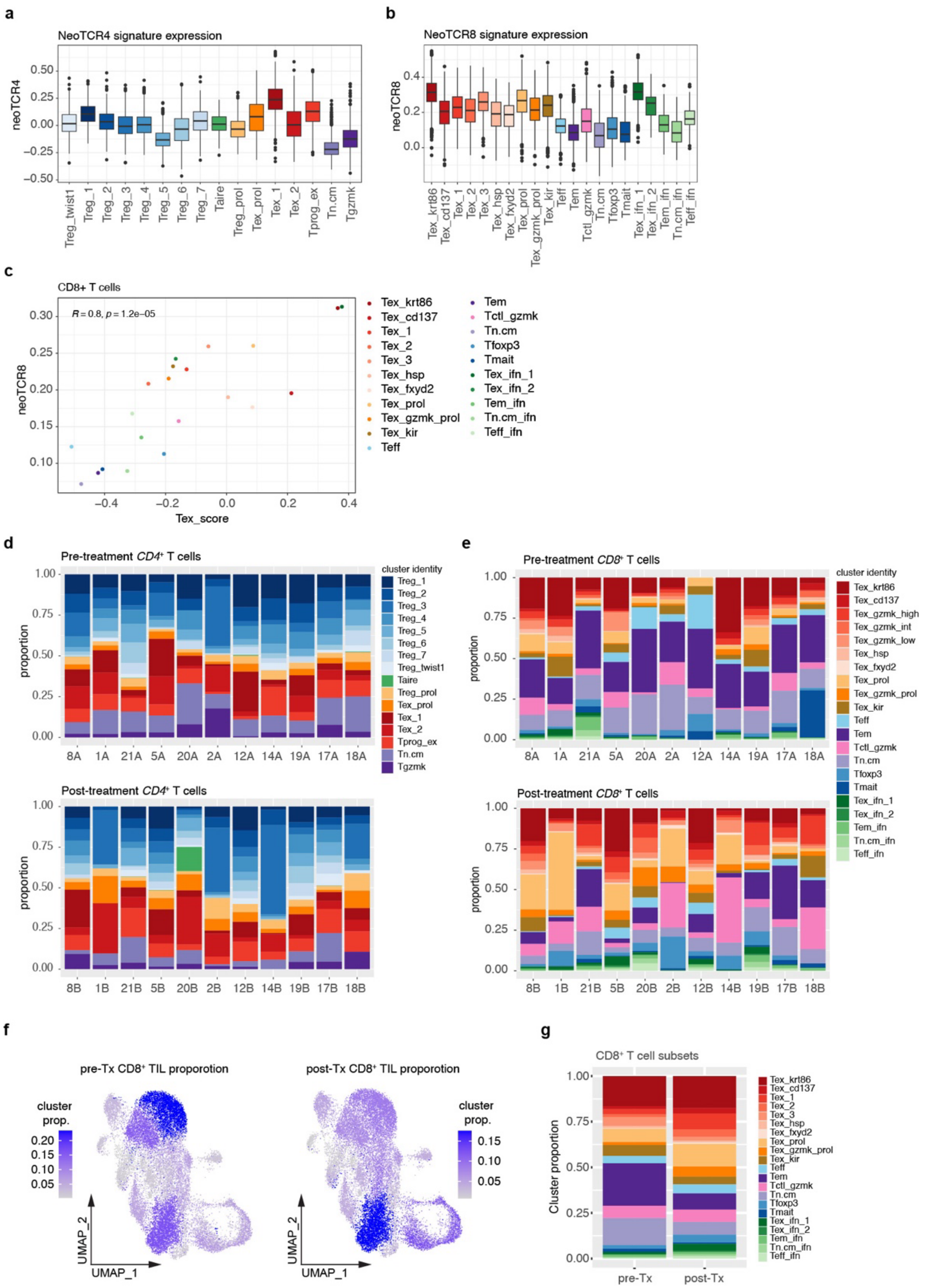

a
NeoTCR4 signature expression
neoTCR4
Treg_twist1
Treg_1
Treg_2
Treg_3
Treg_4
Treg_5
Treg_6
Treg_7
Taire
Treg_prol
Tex_prol
Tex_1
Tex_2
Tprog_ex
Tn.cm
Tgzmk
b
NeoTCR8 signature expression
neoTCR8
Tex_krt86
Tex_cd137
Tex_1
Tex_2
Tex_3
Tex_hsp
Tex_fxyd2
Tex_prol
Tex_gzmk_prol
Tex_kir
Teff
Tem
Tctl_gzmk
Tn.cm
Tfoxp3
Tmait
Tex_ifn_1
Tex_ifn_2
Tem_ifn
Tn.cm_ifn
Teff_ifn
c
CD8+ T cells
R = 0.8, p = 1.2e−05
neoTCR8
Tex_score
d
Pre-treatment CD4+ T cells
Post-treatment CD4+ T cells
proportion
cluster identity
Treg_1
Treg_2
Treg_3
Treg_4
Treg_5
Treg_6
Treg_7
Treg_twist1
Taire
Treg_prol
Tex_prol
Tex_1
Tex_2
Tprog_ex
Tn.cm
Tgzmk
8A 1A 21A 5A 20A 2A 12A 14A 19A 17A 18A
8B 1B 21B 5B 20B 2B 12B 14B 19B 17B 18B
e
Pre-treatment CD8+ T cells
Post-treatment CD8+ T cells
proportion
cluster identity
Tex_krt86
Tex_cd137
Tex_gzmk_high
Tex_gzmk_int
Tex_gzmk_low
Tex_hsp
Tex_fxyd2
Tex_prol
Tex_gzmk_prol
Tex_kir
Teff
Tem
Tctl_gzmk
Tn.cm
Tfoxp3
Tmait
Tex_ifn_1
Tex_ifn_2
Tem_ifn
Tn.cm_ifn
Teff_ifn
f
pre-Tx CD8+ TIL proporotion
post-Tx CD8+ TIL proporotion
cluster prop.
UMAP_1
UMAP_2
g
CD8+ T cell subsets
Cluster proportion
pre-Tx
post-Tx

Figure S3. NeoTCR signature expression and sample distribution across T cell phenotypes.
a, b, Box plots showing the expression scores of the NeoTCR4 and the NeoTCR8 signatures in (e) CD4$^+$- and (f) CD8$^+$ T cell subsets, respectively.
c, Scatter plot showing the mean T-cell exhaustion – and the NeoTCR8 signature scores computed for individual CD8$^+$ T cell subsets. The T-cell exhaustion signature encompasses the following genes: TOX, CTLA4, TIGIT, PDCD1, LAG3, HAVCR2, CXCL13, ENTPD1. P-values are based on Fisher's Z-transform.
d, e, Stacked bar graphs showing the proportions of (a) *CD4*$^+$ and (b) *CD8*$^+$ T cell phenotypes for each pre- (top) and post-Tx (bottom) sample.
f, Scatter plot showing UMAP embeddings of *CD8*$^+$ T cells colored by proportions of respective T cell phenotypes within both pre- (left) and post-treatment cells (right).
g, Stacked bar graph showing the proportions of *CD8*$^+$ T cell phenotypes considering pre- and post-Tx cells.

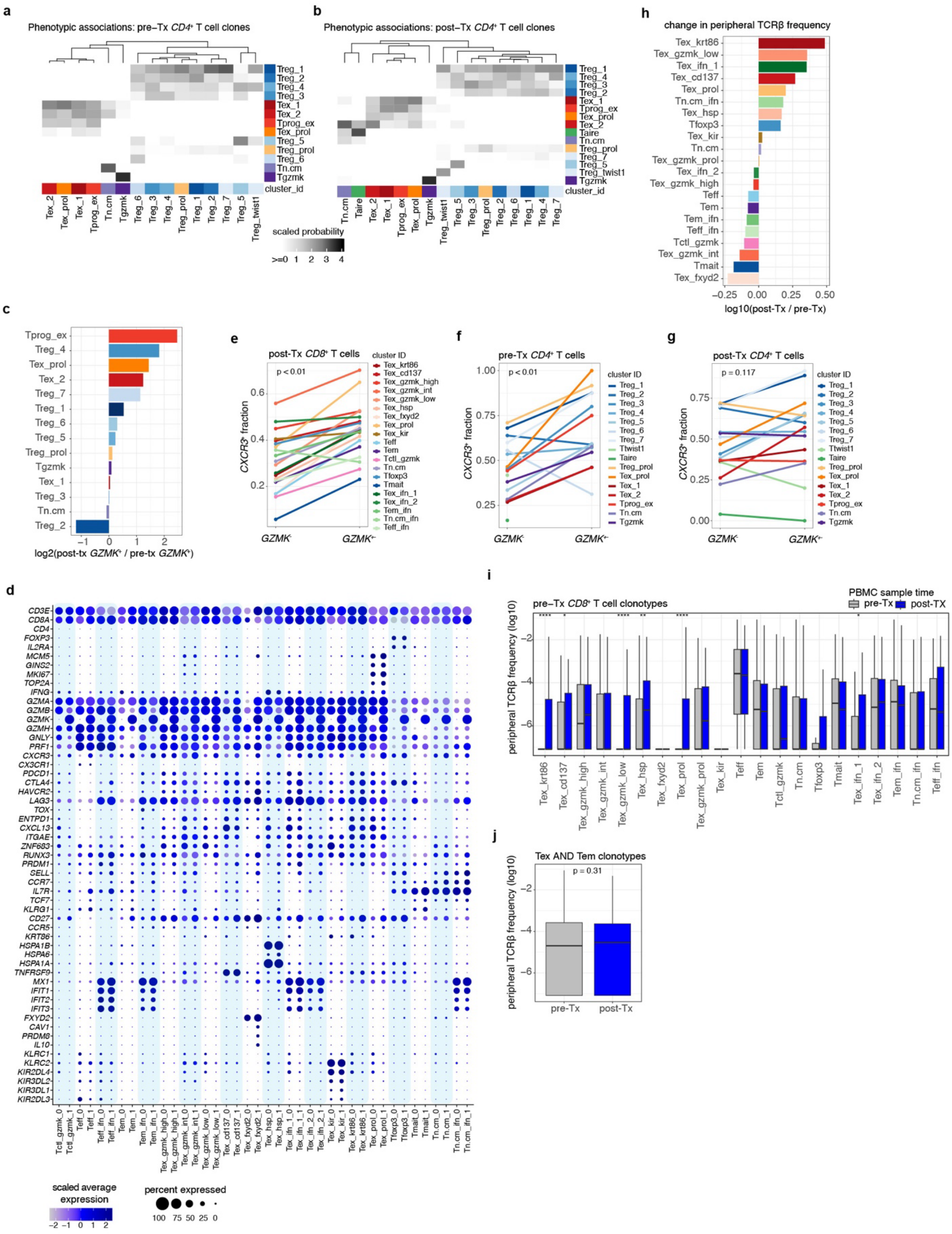
a
Phenotypic associations: pre–Tx CD4+ T cell clones
b
Phenotypic associations: post–Tx CD4+ T cell clones
cluster_id
scaled probability
>=0 1 2 3 4
h
change in peripheral TCRβ frequency
log10(post-Tx / pre-Tx)
c
log2(post-tx GZMK+ / pre-tx GZMK+)
e
post-Tx CD8+ T cells
f
pre-Tx CD4+ T cells
g
post-Tx CD4+ T cells
CXCR3+ fraction
cluster ID
p < 0.01
p < 0.01
p = 0.117
GZMK-
GZMK+
d
scaled average expression
percent expressed
100 75 50 25 0
i
pre–Tx CD8+ T cell clonotypes
PBMC sample time
pre-Tx
post-TX
peripheral TCRβ frequency (log10)
j
Tex AND Tem clonotypes
p = 0.31
peripheral TCRβ frequency (log10)
pre-Tx
post-Tx

Figure S4. Phenotypic and peripheral frequency changes of tumor-infiltrating T cells subsets.

a, b, Heatmaps showing column-standardized, weighted averages of probabilities of two phenotypes co-occurring in a *CD4*$^{+}$ T cell clone in both (a) pre-Tx and (b) post-Tx T cells. Clone-based probability estimates were multiplied by the normalized log10-transformed clone size to account for differences in clone size.

c, Bar graph showing the log2-transformed fold change in the fraction of *GZMK*-positive pre- and post-Tx T cells for different *CD4*$^{+}$ T cell phenotypes.

d, Dot plot showing scaled average expression of T cell marker genes in CD8+ T cells associated with different T cell phenotypes that are further divided into *GZMK*-negative and *GZMK*-positive subsets, indicted by the suffix 0 or 1, respectively. Circle size corresponds to fraction of cells with detectable expression for a given gene.

e, Line graphs showing the fraction of *CXCR3*-positive post-Tx *CD8*$^{+}$ T cells associated with different phenotypes that are distinguished based on the presence or absence of *GZMK* expression. P-value; paired Wilcoxon rank-sum test.

f, g, Line graphs showing the fraction of *CXCR3*-positive (f) pre- or (g) post-Tx *CD4*$^{+}$ T cells associated with different phenotypes that are distinguished based on the presence or absence of *CD8*$^{+}$ expression. P-value; paired Wilcoxon rank-sum test.

h, Bar graph showing the log10-transformed fold change in mean peripheral TCRβ frequencies of clonotypes associated with indicated phenotypes.

i, Box plot showing pre- and post-Tx peripheral TCRβ frequencies of pre-Tx *CD8*$^{+}$ T cell clonotypes associated with indicated phenotypes. P-value; paired Wilcoxon rank-sum test; *: 0.05 >= p-value > 0.01; **: 0.01 >= p-value > 0.001; ***: 0.001 >= p-value > 0.0001.

j, Box plot showing pre- and post-Tx peripheral TCRβ frequencies of *CD8*$^{+}$ Tem/Tex clonotypes. P-value; Wilcoxon rank-sum test.

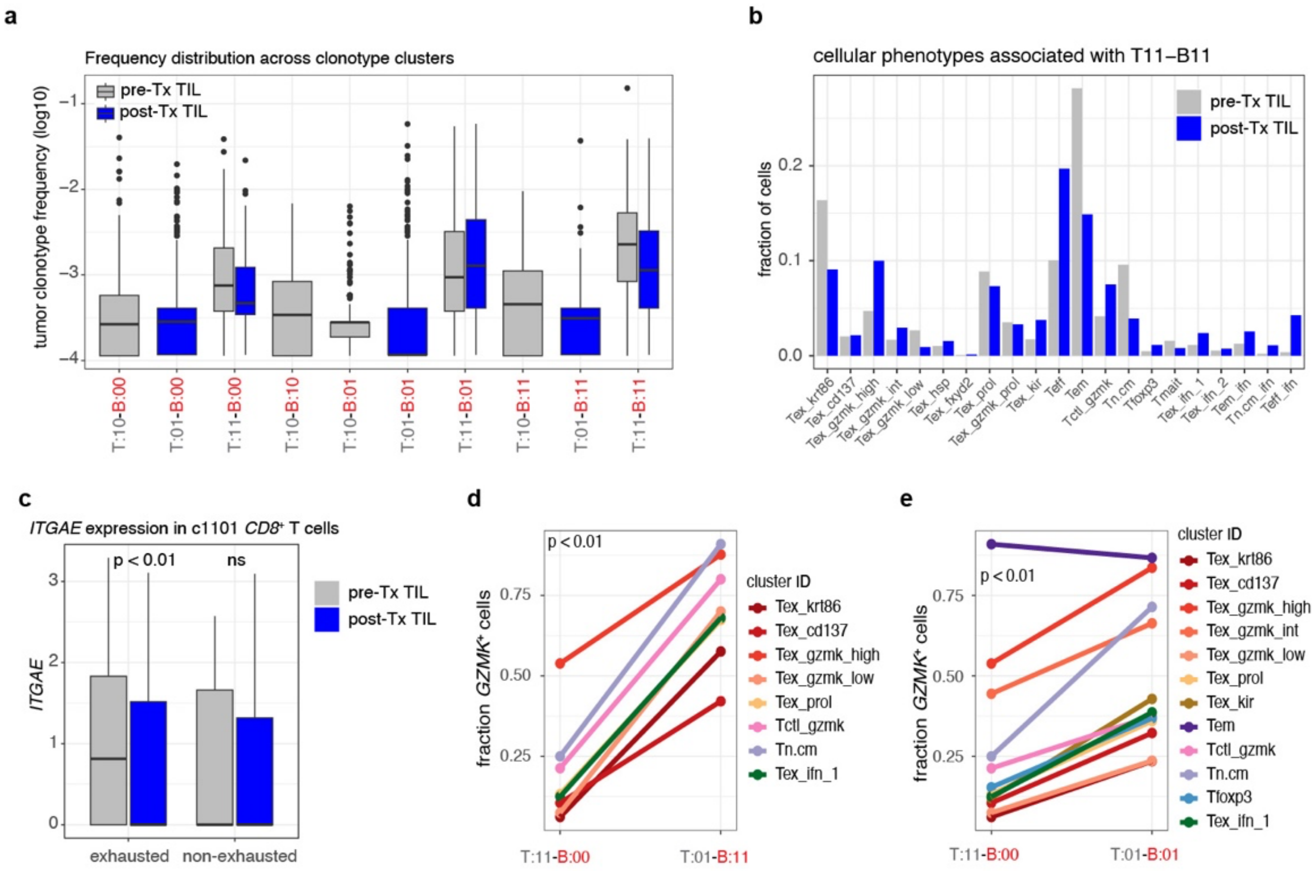

Figure S5. Characterization of persistent and emergent properties associated with distinct clonotype classes.

a, Box plot shows log10-transformed pre- and post-Tx clonotype frequencies of clonotypes associated with different clonotype classes.

b, Bar graph showing the fraction of pre- and post-Tx *CD8*$^{+}$ T cells associated with the T:11-B:11 clonotype class assigned to indicated phenotypes.

c, Box plot showing *ITGAE* expression in exhausted and non-exhausted *CD8*$^{+}$ T cells associated with T:11-B:01 clonotype class pre- and post-Tx. P-value; Wilcoxon rank-sum test.

d, e, Line graphs showing the fraction of *GZMK*-positive post-Tx *CD8*$^{+}$ T cells within the pre-Tx tumor confined clonotype class T:11-B:00 and the tumor emergent clonotype classes (d) T:01-B:11 or (e) T:01-B:01 for the indicated phenotypes. P-value; Wilcoxon rank-sum test.

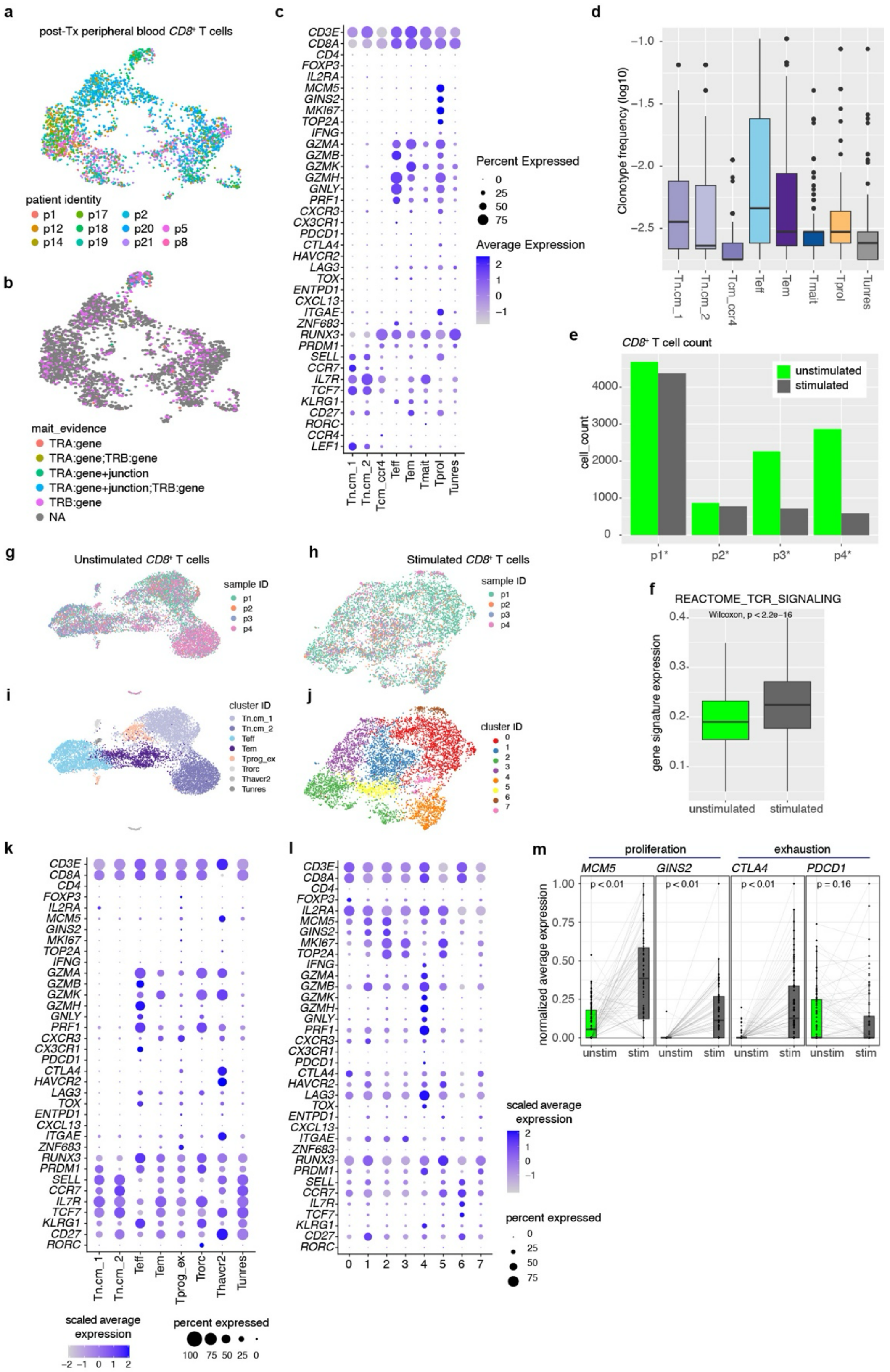

a
post-Tx peripheral blood CD8+ T cells
patient identity
p1 p17 p2
p12 p18 p20 p5
p14 p19 p21 p8
b
mait_evidence
TRA:gene
TRA:gene;TRB:gene
TRA:gene+junction
TRA:gene+junction;TRB:gene
TRB:gene
NA
c
CD3E
CD8A
CD4
FOXP3
IL2RA
MCM5
GINS2
MKI67
TOP2A
IFNG
GZMA
GZMB
GZMK
GZMH
GNLY
PRF1
CXCR3
CX3CR1
PDCD1
CTLA4
HAVCR2
LAG3
TOX
ENTPD1
CXCL13
ITGAE
ZNF683
RUNX3
PRDM1
SELL
CCR7
IL7R
TCF7
KLRG1
CD27
RORC
CCR4
LEF1
Tn.cm_1 Tn.cm_2 Tcm_ccr4 Teff Tem Tmait Tprol Tunres
Percent Expressed
0 25 50 75
Average Expression
2 1 0 −1
d
Clonotype frequency (log10)
−1.0 −1.5 −2.0 −2.5
Tn.cm_1 Tn.cm_2 Tcm_ccr4 Teff Tem Tmait Tprol Tunres
e
CD8+ T cell count
cell_count
4000 3000 2000 1000 0
unstimulated
stimulated
p1* p2* p3* p4*
f
REACTOME_TCR_SIGNALING
Wilcoxon, p < 2.2e-16
gene signature expression
0.4 0.3 0.2 0.1
unstimulated stimulated
g
Unstimulated CD8+ T cells
sample ID
p1 p2 p3 p4
h
Stimulated CD8+ T cells
sample ID
p1 p2 p3 p4
i
cluster ID
Tn.cm_1 Tn.cm_2 Teff Tem Tprog_ex Trorc Thavcr2 Tunres
j
cluster ID
0 1 2 3 4 5 6 7
k
CD3E
CD8A
CD4
FOXP3
IL2RA
MCM5
GINS2
MKI67
TOP2A
IFNG
GZMA
GZMB
GZMK
GZMH
GNLY
PRF1
CXCR3
CX3CR1
PDCD1
CTLA4
HAVCR2
LAG3
TOX
ENTPD1
CXCL13
ITGAE
ZNF683
RUNX3
PRDM1
SELL
CCR7
IL7R
TCF7
KLRG1
CD27
RORC
Tn.cm_1 Tn.cm_2 Teff Tem Tprog_ex Trorc Thavcr2 Tunres
scaled average expression
−2 −1 0 1 2
percent expressed
100 75 50 25 0
l
CD3E
CD8A
CD4
FOXP3
IL2RA
MCM5
GINS2
MKI67
TOP2A
IFNG
GZMA
GZMB
GZMK
GZMH
GNLY
PRF1
CXCR3
CX3CR1
PDCD1
CTLA4
HAVCR2
LAG3
TOX
ENTPD1
CXCL13
ITGAE
ZNF683
RUNX3
PRDM1
SELL
CCR7
IL7R
TCF7
KLRG1
CD27
RORC
0 1 2 3 4 5 6 7
scaled average expression
2 1 0 −1
percent expressed
0 25 50 75
m
proliferation
exhaustion
MCM5 GINS2 CTLA4 PDCD1
p < 0.01 p < 0.01 p < 0.01 p = 0.16
normalized average expression
1.00 0.75 0.50 0.25 0.00
unstim stim unstim stim unstim stim unstim stim

Figure S6. Characterization of *GZMK*-expression within peripheral blood *CD8*$^+$ T cells.

a, Scatter plots showing UMAP embedding of post-Tx peripheral-blood *CD8*$^+$ T cells colored by patient identity.

b, Scatter plots showing UMAP embedding of post-Tx peripheral-blood *CD8*$^+$ T cells colored by MAIT evidence.

c, Dot plot showing scaled average expression of T cell marker genes in post-Tx peripheral-blood *CD8*$^+$ T cells within indicated T cell subsets. Circle size corresponds to fraction of cells with detectable expression for a given gene.

d, Box plot showing the log10-transformed clonotype frequencies of clonotypes associated with the indicated post-Tx peripheral-blood *CD8*$^+$ T cell phenotypes.

e, Bar graph showing the total number of blood-derived scRNA/VDJ-sequenced *CD8*$^+$ T cells isolated from blood samples of four independent HNSCC patients. The corresponding T cells were cultured with or without TCR stimulation (anti-CD28/CD3) and subjected to scRNA/VDJ-seq.

f, Box plot showing expression of a TCR signaling gene expression signature within unstimulated and stimulated blood-derived *CD8*$^+$ T cells. P-value; Wilcoxon rank-sum test.

g, h, i, j, Scatter plots showing UMAP embedding of *CD8*$^+$ T cells isolated from peripheral blood samples of four additional patients not included elsewhere in this study. The corresponding T cells were cultured in (g, i) absence or (h, j) presence of TCR stimulation (anti-CD28/CD3). Colors correspond to (g, i) cluster or (h, j) patient identity.

k, l, Dot plots showing scaled average expression of T cell marker genes in (k) unstimulated and (l) TCR-stimulated peripheral blood-derived CD8+ T cells within indicated T cell subsets. Circle size corresponds to fraction of cells with detectable expression for a given gene.

m, Box plots showing clonotype-average gene expression of peripheral blood-derived *CD8*$^+$ T cells associated with Tem clonotypes, i.e. all clonotypes with at least two Tem cells and non-zero GZMK expression, comparing unstimulated and TCR stimulated T cells. Only clonotypes shared between experimental condition were considered. P-value; paired Wilcoxon rank-sum test.

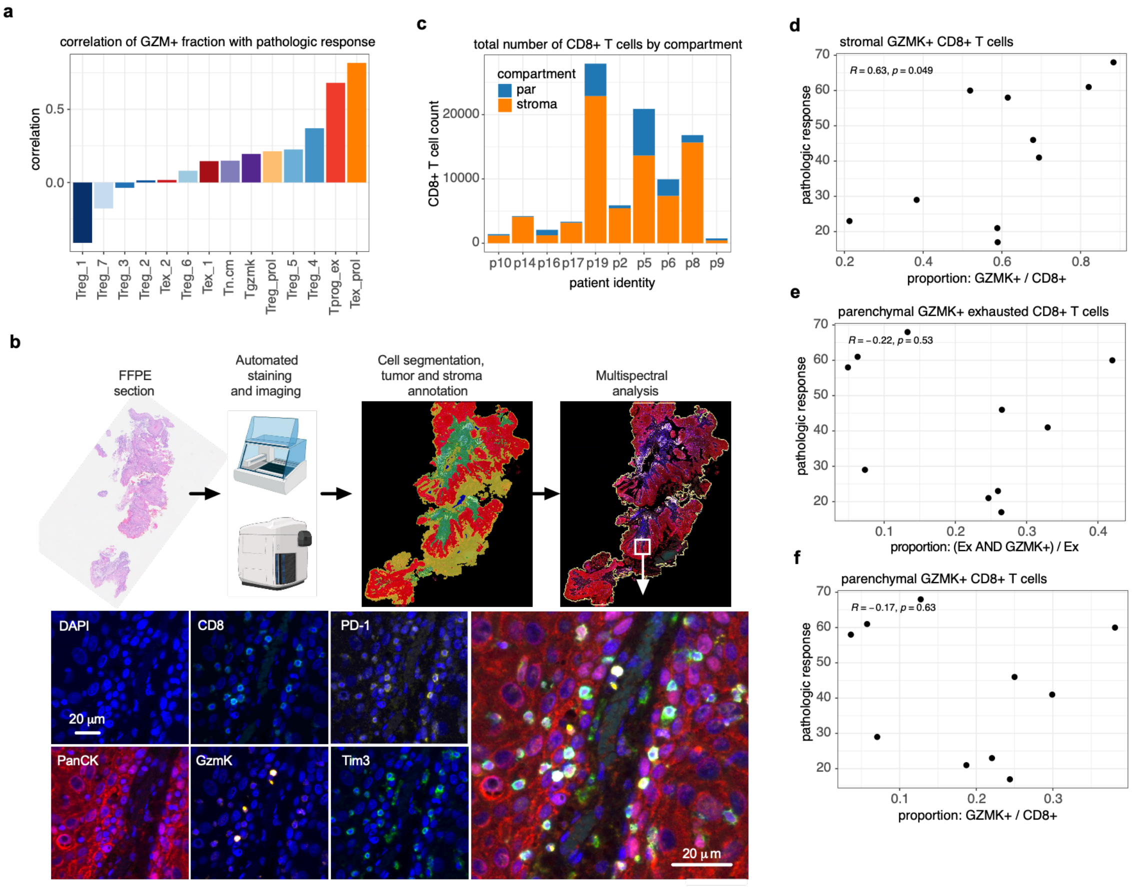

Figure S7. Associations of TIL properties with pathologic responses.

a, Bar graph showing the correlation of *GZMK*-positivity within *CD4*$^{+}$ T cell subsets and pathologic response.

b, Schematic illustrating the workflow used to generate and analyze multiplex immunofluorescence data, along with representative micrographs of individual and combination staining.

c, Stacked bar graph showing the total number of CD8^{+} T cells classified as stromal or parenchymal using multiplex IHC data.

d, Scatter plot showing proportion of GZMK-positive CD8^{+} T cells relative to all CD8^{+} T cells within the stroma and corresponding pathologic responses. Proportions are based on exhaustion panel multiplex IHC data. P-values are based on Fisher's Z-transform.

e, Scatter plot showing proportion of GZMK-positive and exhausted CD8^{+} T cells compared to all exhausted CD8^{+} T cells within the parenchyma and corresponding pathologic responses.

Proportions are based on exhaustion panel multiplex IHC data. P-values are based on Fisher's Z-transform.

f, Scatter plot showing proportion of GZMK-positive and exhausted $CD8^+$ T cells compared to all exhausted $CD8^+$ T cells within the parenchyma and corresponding pathologic responses. Proportions are based on exhaustion panel multiplex IHC data. P-values are based on Fisher's Z-transform.

Table S1. Patient characteristics and pathologic responses.

Patient_ID	Age (years)	Sex	Primary tumor site	Clinical TNM[a]	Pathologic TNM	Treatment arm[b]	Pathologic response (% regression)
1	72	M	OC[c]	T3N2b	T4aN2b	1	27
2	75	F	OC	T3N2b	T4aN0	1	41
3	70	M	OC	OC T4aN0	T2N0	1	70
4	40	M	OC	T4aN2b	T4aN2b	1	6
5	48	F	OC	T4bN2b	T4aN3b	1	29
6	44	M	OC	T4aN2b	T3N0	1	61
7	53	F	OC	T3N0	T3N3b	1	62
8	69	F	OC	T3N2c	T3N0	1	23
9	69	M	OC	T3N1	T4aN0	1	21
10	57	M	OC	T2N0	T1N0	1	17
11	79	M	OC	T2N0	T1N0	1	50
12	46	M	OC	T2N2b	T1N0	1	43
13	41	F	OC	T3N2b	T2N2b	1	68
14	64	F	OC	T4N2c	T3N3b	1	46
16	57	F	larynx	T4aN0	T4aN1	2	58
17	48	F	OC	T2N2b	T2N1	2	68
18	32	F	OC	T3N2c	T2N0	2	89
19	81	F	OC	T3N2b	T2N3b	2	60
20	69	F	OC	T4aN2b	T4aN0	2	31
21	70	M	OC	T2N0	T2N0	2	27

[a]T, tumor; N, nodal disease
[b]Arm 1 = bintrafusp alfa; Arm 2 = bintrafusp alfa plus Tri-Ad5
[c]OC, oral cavity

Table S2. Patient level summary of clinical specimen use and data availability.

Patient_ID	Deep TCR-seq peripheral T cells		scRNA/VDJ-seq peripheral T cells	scRNA/VDJ-seq TIL		IF panel 1	IF panel 2	
	Pre-Tx	Post-Tx	Post-Tx	Pre-Tx	Post-Tx	Pre-Tx	Pre-Tx	Post-Tx
1	yes	yes	yes	yes	yes		yes	yes
2	yes	yes	yes	yes	yes	yes	yes	yes
3							yes	yes
4								
5	yes	yes	yes	yes	yes	yes	yes	yes
6						yes	yes	yes
7								
8	yes	yes	yes	yes	yes	yes	yes	yes
9						yes	yes	yes
10						yes	yes	yes
11								
12	yes	yes	yes	yes	yes		yes	yes
13							yes	yes
14	yes	yes	yes	yes	yes	yes	yes	yes
16						yes	yes	yes
17	yes	yes	yes	yes	yes	yes	yes	yes
18	yes	yes	yes	yes	yes			
19	yes	yes	yes	yes	yes	yes	yes	yes
20	yes	yes	yes	yes	yes		yes	yes
21	yes	yes	yes	yes	yes		yes	yes

IF (immunofluorescence) panel 1 = DAPI, pan-cytokeratin, CD8, granzyme K, PD-1, Tim3
IF panel 2 = DAPI, pan-cytokeratin, CD8, granzyme K, TCF7, Ki67

Table S3. Top ten upregulated genes for each CD8$^+$ T cell subsets. A positive log2 fold change indicates upregulation in the corresponding T-cell cluster. Pct.1 and pct.2 indicate proportion of cells with non-zero gene expression within the indicated cluster and the control cells, respectively.

p_val	avg_log2FC	pct.1	pct.2	p_val_adj	cluster	gene
0	2.318666444282265	0.837	0.237	0	Tex_1	GZMK
5.8804737678398934e-210	0.4695784954265827	0.999	0.999	2.15231220376708e-205	Tex_1	RPS15A
2.1195882200320907e-143	0.3881917421718555	1	0.999	7.757904844139456e-139	Tex_1	RPL28
2.401910624204046e-142	0.81243043	0.944	0.776	8.791233075649227e-138	Tex_1	GZMH
4.520721440568341e-127	0.8053862495944202	0.949	0.817	1.6546292544624184e-122	Tex_1	CXCR4
4.905122681505957e-122	0.7167615685856172	0.954	0.831	1.7953239526579948e-117	Tex_1	CST7
8.104796511853972e-119	1.3043350549142203	0.613	0.354	2.9664365713036727e-114	Tex_1	GIMAP4
4.0168973152996605e-114	1.3665414955579314	0.574	0.322	1.470224586372829e-109	Tex_1	LIME1
3.272602022463602e-101	0.8424630622334917	0.882	0.713	1.1978050662419028e-96	Tex_1	DUSP2
6.552890721723958e-101	2.0468505318628902	0.253	0.081	2.3984235330581857e-96	Tex_1	LINC00861
3.571266910949239e-108	0.6890066044741738	1	0.975	1.3071194020765308e-103	Tex_2	RPLP0
2.002924775975439e-98	0.4580960838151418	0.999	0.996	7.330904972547702e-94	Tex_2	EEF1A1
2.081585724437977e-98	0.4426010741886319	1	0.997	7.618811910015439e-94	Tex_2	RPL13
1.1921606190772478e-94	0.50255151	0.999	0.986	4.363427081884635e-90	Tex_2	RPS8
5.867558982624941e-94	1.0637812362434262	0.372	0.102	2.147585263230555e-89	Tex_2	KLF2
4.3029777282058177e-91	0.5587991170295128	1	0.992	1.5749328783006114e-86	Tex_2	RPS18
2.7161693823319955e-90	0.6494642921117735	0.999	0.942	9.941451556273335e-86	Tex_2	RPL3
4.02925851043119e-86	1.798113510824845	0.239	0.051	1.4747489074029199e-81	Tex_2	KLRG1
3.6687561150364307e-81	2.6074890271066486	0.114	0.013	1.3428014256644841e-76	Tex_2	PLAC8
2.2888265540340188e-79	1.0487983801641143	0.524	0.192	8.377334070419911e-75	Tex_2	LYAR
1.9634536993960105e-108	0.32730572181882245	1	1	7.186436885159338e-104	Tex_3	B2M
6.4178691075124874e-108	0.5402426293648501	1	0.996	2.349004272040646e-103	Tex_3	SH3BGRL3
3.2968502671377505e-72	0.3832903496972815	1	0.998	1.206680166275088e-67	Tex_3	RPL30
4.0751369198015344e-60	0.5659576866213296	0.986	0.969	1.4915408640165596e-55	Tex_3	S100A4
5.2524828482085275e-51	0.31339335	0.999	0.997	1.9224612472728032e-46	Tex_3	RPL11
9.772565487225985e-50	0.29989164697550663	0.998	0.998	3.5768566939795825e-45	Tex_3	RPS27
2.8012357191552656e-49	0.2627189519718618	1	1	1.0252802855680188e-44	Tex_3	RPL41
5.9236117013285616e-46	0.43952615	0.976	0.977	2.1681011188032663e-41	Tex_3	CD3D
5.978167696814518e-46	0.29844597082209745	0.997	0.996	2.188069158711081e-41	Tex_3	RPL19
8.050600262289478e-46	0.5297143176777839	0.945	0.946	2.946600202000572e-41	Tex_3	S100A6
2.888603974057481e-211	3.715031538825228	0.55	0.119	1.0572579405447787e-206	Tex_cd137	XCL1
2.867164473428506e-199	4.185600495273866	0.498	0.103	1.0494108689195675e-194	Tex_cd137	XCL2
7.828730069456695e-192	2.8848497351039586	0.678	0.214	2.8653934927218445e-187	Tex_cd137	CRTAM

3.8907493410218716e-158	2.264240706041425	0.719	0.279	1.4240531663074154e-153	Tex_cd137	TNFRSF9
1.414706836004581e-150	4.437717982103182	0.148	0.01	5.177968490460366e-146	Tex_cd137	GNG4
2.52842314937294e-99	5.107950689118306	0.084	0.005	9.254281569019898e-95	Tex_cd137	EBI3
1.6916821483038843e-90	1.2565582565738014	0.912	0.751	6.1917258310070466e-86	Tex_cd137	HSP90AB1
1.9554723027822054e-82	1.124721223257886	0.864	0.673	7.157224175413151e-78	Tex_cd137	PKM
4.659653894659496e-82	2.3729152654348966	0.383	0.119	1.7054799219843226e-77	Tex_cd137	MIR155HG
4.4180564231362624e-76	1.888151659640538	0.538	0.245	1.6170528314321033e-71	Tex_cd137	NME1
0	6.878171054302032	0.778	0.028	0	Tex_fxyd2	FXYD2
0	7.032916422197458	0.278	0.003	0	Tex_fxyd2	CAV1
0	9.022311652929746	0.159	0.001	0	Tex_fxyd2	ANKRD55
6.905278522184021e-273	7.902520833401342	0.159	0.001	2.527400991904574e-268	Tex_fxyd2	MIR4422HG
7.269989899763918e-240	8.485354165797206	0.135	0.001	2.660889003212592e-235	Tex_fxyd2	PTPN3
1.2989788029208604e-141	10.2543721	0.048	0	4.754392316570642e-137	Tex_fxyd2	COL19A1
5.676037886377923e-123	4.498330672665846	0.46	0.039	2.077486626793184e-118	Tex_fxyd2	CADM1
1.2277532649064395e-121	4.484451833294768	0.286	0.015	4.4936997248840585e-117	Tex_fxyd2	IL10
2.26328163412651e-116	4.857342662910642	0.262	0.013	8.283837109066442e-112	Tex_fxyd2	PRDM8
4.012484686332361e-115	5.7207197217662875	0.183	0.006	1.4686095200445078e-110	Tex_fxyd2	ADTRP
0	1.27810779	1	0.25	0	Tex_gzmk_prol	GZMK
0	2.265035708305801	0.827	0.192	0	Tex_gzmk_prol	TYMS
0	2.0395728695934676	0.964	0.343	0	Tex_gzmk_prol	STMN1
0	2.246892909110387	0.754	0.163	0	Tex_gzmk_prol	PCLAF
0	2.7165056166643953	0.574	0.096	0	Tex_gzmk_prol	RRM2
1.528536141e-298	2.481995161127734	0.508	0.087	5.594595129673e-295	Tex_gzmk_prol	UBE2C
2.9525276721838007e-291	2.2934260421812844	0.523	0.092	1.0806546532959928e-291	Tex_gzmk_prol	ZWINT
6.304313541526136e-290	2.387755541325194	0.46	0.075	2.307441799333981e-285	Tex_gzmk_prol	ASF1B
2.1288398716397695e-287	2.101505541598126	0.556	0.11	7.79176681418872e-283	Tex_gzmk_prol	CENPW
2.902857172414981e-287	2.2349565903962065	0.525	0.099	1.062474753675607e-282	Tex_gzmk_prol	CENPU
0	6.668601182455738	0.912	0.061	0	Tex_hsp	HSPA1B
0	8.625415794082185	0.268	0.004	0	Tex_hsp	HSPA6
4.73098307760821e-286	6.070765202334911	0.967	0.184	1.7315871162353814e-281	Tex_hsp	HSPA1A
7.220806148534073e-164	4.575460379300544	0.979	0.426	2.642887258424955e-159	Tex_hsp	DNAJB1
1.899519473843475e-113	3.960279513704577	0.695	0.202	6.952431226214504e-109	Tex_hsp	HSPH1
6.6655140421872704e-108	4.449966235585858	0.418	0.066	2.4396447945809626e-103	Tex_hsp	BAG3
5.151362602650968e-107	5.609390423445788	0.163	0.009	1.8854502261962806e-102	Tex_hsp	SERPINH1
5.965127547961532e-92	2.9231328438424344	0.958	0.817	2.1832963338294e-87	Tex_hsp	HSP90AA1
4.4098477865031305e-81	4.412333645283585	0.238	0.027	1.6140483883380112e-76	Tex_hsp	DNAJB4
6.734562458248505e-76	1.9250515162098707	0.958	0.872	2.4649172053435357e-71	Tex_hsp	HSPA8
0	4.245481131927914	0.683	0.067	0	Tex_ifn_1	IFIT1

0	3.7712802489550032	0.577	0.078	0	Tex_ifn_1	RSAD2
0	4.360573923295925	0.535	0.049	0	Tex_ifn_1	IFIT3
1.8053e-303	4.442763783981534	0.361	0.03	6.6076366e-300	Tex_ifn_1	IFIT2
1.3573433131834676e-272	3.170677549632296	0.611	0.119	4.96801226058281e-268	Tex_ifn_1	HERC5
5.86246157360824e-250	2.7072073795876537	0.851	0.323	2.145719560556352e-245	Tex_ifn_1	MX1
4.072039632805814e-235	2.2450707427053525	0.97	0.659	1.4904072260032557e-230	Tex_ifn_1	ISG15
2.1885549111580286e-230	2.79109704	0.726	0.219	8.0103298303295e-226	Tex_ifn_1	OAS1
1.2179943364598046e-215	3.1958694092295463	0.512	0.102	4.45798107087653e-211	Tex_ifn_1	MX2
3.407227281678734e-203	2.2533385111482156	0.913	0.502	1.2470792573672331e-198	Tex_ifn_1	IFI6
3.158317804548941e-85	2.975465161704835	0.56	0.088	1.155975899642958e-80	Tex_ifn_2	IFIT1
1.2592913633661155e-67	3.0466867704209415	0.433	0.066	4.60913231905632e-63	Tex_ifn_2	IFIT3
7.231510803744135e-57	2.0461084412732444	0.979	0.669	2.646805269278391e-52	Tex_ifn_2	ISG15
8.225021137216343e-57	2.1870514708382487	0.752	0.235	3.010439986432553e-52	Tex_ifn_2	OAS1
1.2897388142911363e-54	1.6099832371379614	0.993	0.766	4.720573034186988e-50	Tex_ifn_2	ISG20
1.3022105054161384e-52	1.3488557244150483	1	0.966	4.766220670873608e-48	Tex_ifn_2	IFITM1
8.614091958156493e-44	2.395738274758229	0.482	0.115	3.152843797604859e-39	Tex_ifn_2	MX2
2.439500534983269e-40	2.3788058017364504	0.426	0.096	8.928815908092263e-36	Tex_ifn_2	RSAD2
2.771867858618784e-40	1.773252616073298	0.801	0.34	1.0145313549330612e-35	Tex_ifn_2	MX1
4.0828829986636643e-36	1.576462855860879	0.879	0.515	1.4943760063408879e-31	Tex_ifn_2	IFI6
0	2.369230548671057	0.816	0.226	0	Tex_kir	KLRC2
0	3.674846788892256	0.355	0.04	0	Tex_kir	KIR3DL2
0	4.842738891328484	0.202	0.01	0	Tex_kir	KIR3DL1
3.38354434654089e-257	1.7671654443182372	0.634	0.21	1.238411066277431e-252	Tex_kir	KIR2DL4
1.4622388660165553e-245	4.026782016622201	0.196	0.018	5.351940473507194e-241	Tex_kir	KIR2DL3
1.3588496184423603e-200	4.791242439849476	0.137	0.009	4.973525488460883e-196	Tex_kir	KIR2DL1
1.659960672914033e-196	2.849901991193555	0.257	0.045	6.075622058932652e-192	Tex_kir	LAT2
4.210456680267487e-195	1.2854814703427675	0.798	0.414	1.541069249544703e-190	Tex_kir	HOPX
5.242767991482007e-189	4.185026829194303	0.149	0.013	1.9189055125623292e-184	Tex_kir	TYROBP
2.2282569444946957e-178	2.828701376785691	0.243	0.044	8.155643242545036e-174	Tex_kir	KLRC3
5.5129091258212894e-282	1.2198532992896285	0.717	0.479	2.01777986914185e-277	Tex_krt86	CXCR6
1.246083114837273e-232	0.8838242625327544	0.862	0.731	4.560788808615904e-228	Tex_krt86	LINC01871
1.9339163939334444e-206	0.5812558418592801	0.97	0.928	7.078327393435799e-202	Tex_krt86	CD7
1.0808550316881192e-171	1.892147797364148	0.345	0.15	3.956037501481685e-167	Tex_krt86	KRT86
5.297069107640052e-170	0.8715633373228151	0.762	0.641	1.9387802640873358e-165	Tex_krt86	TIGIT
2.7333956791906953e-165	0.20827212828069097	1	1	1.0004501525405864e-160	Tex_krt86	B2M
8.285320383636881e-160	1.622608401082048	0.341	0.151	3.032510113614935e-155	Tex_krt86	KLRB1
2.715482730583902e-159	0.7145682625982737	0.846	0.747	9.93893834221014e-155	Tex_krt86	RGS1
1.068062369063839e-157	1.020591826319479	0.646	0.491	3.9092150770105575e-153	Tex_krt86	ACP5

6.917913664502438e-157	1.475638504592396	0.4	0.21	2.5320255803445368e-152	Tex_krt86	LAYN
0	3.000912005901033	0.905	0.268	0	Tex_prol	STMN1
0	3.174921548057715	0.746	0.12	0	Tex_prol	TYMS
0	3.03289094	0.63	0.106	0	Tex_prol	PCLAF
0	2.5093902581638323	0.555	0.12	0	Tex_prol	MCM7
0	2.555444730615271	0.825	0.394	0	Tex_prol	TUBA1B
0	2.4783828377714423	0.751	0.323	0	Tex_prol	TUBB
0	2.019026135169758	0.766	0.338	0	Tex_prol	DUT
0	2.2992190985430394	0.567	0.145	0	Tex_prol	PCNA
0	1.8018232665145413	0.639	0.222	0	Tex_prol	NUDT1
0	2.9417640902214592	0.492	0.091	0	Tex_prol	MKI67

Table S4. Patient characteristics. These patients donated peripheral blood T cells, but were not treated on the clinical trial.

Pt_ID	Age (years)	Sex	Primary tumor site[a]	Clinical TNM[b]
PBMC_1	55	M	OC	T4N2b
PBMC_2	66	F	OC	T3N0
PBMC_3	72	M	OC	T3N1
PBMC_4	61	F	OC	T4N2b

[a]OC, oral cavity

[b]T, tumor; N, nodal disease

Table S5. Multispectral immunofluorescence reagents and details

Panel 1

Antibody	Vendor /Clone	Catalog #	Dilution 1:	HIER	Secondary Antibody	Opal	Opal dilution 1:
Tim3	CST[D5D5R]	ab245620	25	Leica ER2(AR9640) / 20 min	BioCare MACH 2 Rabbit HRP-Polymer (RHRP520)	520	150
PD-1	Abcam [EPR4877(2)]	ab137132	750	Leica ER2(AR9640) / 20 min	Akoya OPAL POLYMER HRP MS + RB, 1X, (ARH1001EA)	570	150
CD8	Abcam [EPR10640-2]	ab215041	2000	Leica ER2(AR9640) / 20 min	Akoya OPAL POLYMER HRP MS + RB, 1X, (ARH1001EA)	480	150
Granzyme K	Abcam [EPR24601-164]	ab282703	200	Leica ER2(AR9640) / 20 min	BioCare MACH 2 Rabbit HRP-Polymer (RHRP520)	620	150
Ki67	Ventana [30-9]	790-4286	1	Leica ER1 (AR9961) / 20 min	Akoya OPAL POLYMER HRP MS + RB, 1X, (ARH1001EA)	690	150
PanCK	Santa Cruz [AE1/AE3]	sc-81714	400	Leica ER2(AR9640) / 20 min	Akoya OPAL POLYMER HRP MS + RB, 1X, (ARH1001EA)	780	50

Panel 2

Antibody	Vendor /Clone	Catalog #	Dilution 1:	HIER	Secondary Antibody	Opal	Opal dilution 1:
Granzyme K	Abcam [EPR24601-164]	ab282703	200	Leica ER2(AR9640) / 20 min	BioCare MACH 2 Rabbit HRP-Polymer (RHRP520)	520	150

TCF-7	Abcam [EPR28579-5]	ab315392	200	Leica ER2(AR9640) / 20 min	Akoya OPAL POLYMER HRP MS + RB, 1X, (ARH1001EA)	570	150
CD4	Abcam [EPR6855]	ab133616	1000	Leica ER1 (AR9961) / 20 min	BioCare MACH 2 Rabbit HRP-Polymer (RHRP520)	480	150
CD8	Abcam [EPR10640-2]	ab215041	2000	Leica ER2(AR9640) / 20 min	Akoya OPAL POLYMER HRP MS + RB, 1X, (ARH1001EA)	620	150
Ki67	Ventana [30-9]	790-4286	1	Leica ER1 (AR9961) / 20 min	BioCare MACH 2 Rabbit HRP-Polymer (RHRP520)	690	150
PanCK	Santa Cruz [AE1/AE3]	sc-81714	400	Leica ER2(AR9640) / 20 min	Akoya OPAL POLYMER HRP MS + RB, 1X, (ARH1001EA)	780	50

Table S6. treatment-naïve GZMK signature

gene_id	p_val	avg_log2FC	pct.1*	pct.2*	p_val_adj
GZMK	0	15.6281861	1	0	0
CD27	4.07E-32	1.37395085	0.682	0.309	1.49E-27
LAG3	5.56E-32	1.24346644	0.827	0.492	2.03E-27
TNFRSF9	2.04E-30	2.1373205	0.405	0.14	7.48E-26
ITM2C	2.17E-30	1.26436778	0.75	0.405	7.96E-26
CD81	4.44E-30	1.30785643	0.673	0.332	1.63E-25
GZMH	5.02E-30	0.99621955	0.9	0.646	1.84E-25
AC034238.1	1.06E-28	3.71416863	0.073	0.005	3.88E-24
CXCL13	3.46E-27	1.58106538	0.586	0.259	1.27E-22
ZBED2	5.39E-26	3.49220443	0.114	0.015	1.97E-21
TNFSF4	1.59E-24	2.42327741	0.241	0.064	5.83E-20
NKG7	4.79E-24	0.65715154	0.995	0.901	1.75E-19
NAB1	6.57E-23	1.88552519	0.314	0.105	2.41E-18
SEMA4A	2.36E-22	2.5690481	0.15	0.029	8.65E-18
TMEM155	4.76E-22	5.01280086	0.041	0.002	1.74E-17
MTRNR2L12	1.71E-21	0.90150013	0.918	0.757	6.26E-17
TSC22D1	2.07E-21	2.24450293	0.191	0.046	7.58E-17
DUSP4	3.07E-21	0.92156994	0.841	0.566	1.13E-16
TOX	3.73E-21	1.66363519	0.332	0.119	1.36E-16
SH2D1A	1.63E-20	1.3324302	0.432	0.187	5.98E-16

APOBEC3G	3.45E-20	0.91246283	0.791	0.523	1.26E-15
VCAM1	1.74E-19	1.79602514	0.395	0.17	6.37E-15
DGKZ	5.86E-19	1.47375917	0.391	0.166	2.14E-14
CRTAM	6.52E-19	1.98847135	0.3	0.109	2.39E-14
ITGB2	1.19E-18	0.90259307	0.664	0.396	4.34E-14
HAVCR2	1.74E-18	1.54222299	0.373	0.157	6.35E-14
LRRN3	1.75E-18	1.70601456	0.186	0.049	6.41E-14
AL391056.1	4.83E-18	5.70318569	0.018	0	1.77E-13
TRBV21-1	8.36E-18	2.45427734	0.109	0.02	3.06E-13
PTMS	1.69E-17	0.87384747	0.686	0.411	6.17E-13
ENPEP	2.43E-17	8.3114285	0.014	0	8.90E-13
HLA-DPA1	4.77E-17	0.64330218	0.905	0.689	1.74E-12
CD44	1.60E-16	0.78086614	0.795	0.569	5.85E-12
SAMSN1	2.02E-16	0.7981414	0.809	0.574	7.38E-12
AL606807.1	3.95E-16	3.16762201	0.055	0.006	1.44E-11
BHLHE40	9.62E-16	0.88224513	0.645	0.389	3.52E-11
IKZF3	9.81E-16	1.00885367	0.541	0.297	3.59E-11
LINC00158	1.14E-15	3.83604049	0.05	0.005	4.17E-11
CD74	2.42E-15	0.51827027	0.995	0.934	8.85E-11
CTLA4	3.04E-15	0.90912879	0.555	0.312	1.11E-10

*pct1, pct2: percent of cells with detectable gene expression in query and reference T cells, respectively.
