# Supplementary material for: GZMK expression within activated intratumoral T-cell subsets reflects differentiation efficiency and predicts response to cancer immunotherapy
Source: NPJ Precis Oncol. 2026 Apr 18;10:237. doi: 10.1038/s41698-026-01437-7 (PMC13280249; doi:10.1038/s41698-026-01437-7)
Supplement: Supplementary file 2 — Supplementary information [file 41698_2026_1437_MOESM2_ESM.docx]

**CONSORT 2025 checklist of information to include when reporting a randomised trial***

| **Section / Topic** | **No** | **CONSORT 2025 checklist item description** | **Reported on page no.** |
| --- | --- | --- | --- |
| **Title and abstract** | | |  |
| Title and structured abstract | 1a | Identification as a randomised trial | n/a – not a randomized trial and trial already reported |
|  | 1b | Structured summary of the trial design, methods, results, and conclusions | n/a – not a randomized trial and trial already reported |
| **Open science** | | |  |
| Trial registration | 2 | Name of trial registry, identifying number (with URL) and date of registration | Page 17 of the manuscript and the abstract |
| Protocol and statistical analysis plan | 3 | Where the trial protocol and statistical analysis plan can be accessed | n/a – not a randomized trial and trial already reported |
| Data sharing | 4 | Where and how the individual de-identified participant data (including data dictionary), statistical code and any other materials can be accessed | All clinical trial design and data information is referenced (to the original clinical trial publications) on page 17 of the manuscript. |
| Funding and conflicts of interest | 5a | Sources of funding and other support (e.g., supply of drugs), and role of funders in the design, conduct, analysis and reporting of the trial | n/a – not a randomized trial and trial already reported |
|  | 5b | Financial and other conflicts of interest of the manuscript authors | All competing interests are declared on page 23 of the manuscript |
| **Introduction** | | |  |
| Background and rationale | 6 | Scientific background and rationale | n/a – not a randomized trial and trial already reported |
| Objectives | 7 | Specific objectives related to benefits and harms | n/a – not a randomized trial and trial already reported |
| **Methods** | | |  |
| Patient and public involvement | 8 | Details of patient or public involvement in the design, conduct and reporting of the trial | n/a – not a randomized trial and trial already reported |
| Trial design | 9 | Description of trial design including type of trial (e.g., parallel group, crossover), allocation ratio, and framework (e.g., superiority, equivalence, non-inferiority, exploratory) | n/a – not a randomized trial and trial already reported |
| Changes to trial protocol | 10 | Important changes to the trial after it commenced including any outcomes or analyses that were not prespecified, with reason | n/a – not a randomized trial and trial already reported |
| Trial setting | 11 | Settings (e.g., community, hospital) and locations (e.g., countries, sites) where the trial was conducted | n/a – not a randomized trial and trial already reported |
| Eligibility criteria | 12a | Eligibility criteria for participants | n/a – not a randomized trial and trial already reported |
|  | 12b | If applicable, eligibility criteria for sites and for individuals delivering the interventions (e.g., surgeons, physiotherapists) | n/a – not a randomized trial and trial already reported |
| Intervention and comparator | 13 | Intervention and comparator with sufficient details to allow replication. If relevant, where additional materials describing the intervention and comparator (e.g., intervention manual) can be accessed | n/a – not a randomized trial and trial already reported |
| Outcomes | 14 | Pre-specified primary and secondary outcomes, including the specific measurement variable (e.g., systolic blood pressure), analysis metric (e.g., change from baseline, final value, time to event), method of aggregation (e.g., median, proportion), and time point for each outcome | n/a – not a randomized trial and trial already reported |
| Harms | 15 | How harms were defined and assessed (e.g., systematically, non-systematically) | n/a – not a randomized trial and trial already reported |
| Sample size | 16a | How sample size was determined, including all assumptions supporting the sample size calculation | n/a – not a randomized trial and trial already reported |
|  | 16b | Explanation of any interim analyses and stopping guidelines | n/a – not a randomized trial and trial already reported |
| Randomisation: |  |  |  |
| Sequence generation | 17a | Who generated the random allocation sequence and the method used | n/a – not a randomized trial and trial already reported |
|  | 17b | Type of randomisation and details of any restriction (e.g., stratification, blocking and block size) | n/a – not a randomized trial and trial already reported |
| Allocation concealment mechanism | 18 | Mechanism used to implement the random allocation sequence (e.g., central computer/telephone; sequentially numbered, opaque, sealed containers), describing any steps to conceal the sequence until interventions were assigned | n/a – not a randomized trial and trial already reported |
| Implementation | 19 | Whether the personnel who enrolled and those who assigned participants to the interventions had access to the random allocation sequence | n/a – not a randomized trial and trial already reported |
| Blinding | 20a | Who was blinded after assignment to interventions (e.g., participants, care providers, outcome assessors, data analysts) | n/a – not a randomized trial and trial already reported |
|  | 20b | If blinded, how blinding was achieved and description of the similarity of interventions | n/a – not a randomized trial and trial already reported |
| Statistical methods | 21a | Statistical methods used to compare groups for primary and secondary outcomes, including harms | n/a – not a randomized trial and trial already reported |
|  | 21b | Definition of who is included in each analysis (e.g., all randomised participants), and in which group | n/a – not a randomized trial and trial already reported |
|  | 21c | How missing data were handled in the analysis | n/a – not a randomized trial and trial already reported |
|  | 21d | Methods for any additional analyses (e.g., subgroup and sensitivity analyses), distinguishing prespecified from post-hoc | n/a – not a randomized trial and trial already reported |
| **Results** | | |  |
| Participant flow, including flow diagram | 22a | For each group, the numbers of participants who were randomly assigned, received intended intervention, and were analysed for the primary outcome | n/a – not a randomized trial and trial already reported |
|  | 22b | For each group, losses and exclusions after randomisation, together with reasons | n/a – not a randomized trial and trial already reported |
| Recruitment | 23a | Dates defining the periods of recruitment and follow-up for outcomes of benefits and harms | n/a – not a randomized trial and trial already reported |
|  | 23b | If relevant, why the trial ended or was stopped | n/a – not a randomized trial and trial already reported |
| Intervention and comparator delivery | 24a | Intervention and comparator as they were actually administered (e.g., where appropriate, who delivered the intervention/comparator, how participants adhered, whether they were delivered as intended [fidelity]) | n/a – not a randomized trial and trial already reported |
|  | 24b | Concomitant care received during the trial for each group | n/a – not a randomized trial and trial already reported |
| Baseline data | 25 | A table showing baseline demographic and clinical characteristics for each group | n/a – not a randomized trial and trial already reported |
| Numbers analysed,  outcomes and estimation | 26 | For each primary and secondary outcome, by group:   - the number of participants included in the analysis - the number of participants with available data at the outcome time point - result for each group, and the estimated effect size and its precision (such as 95% confidence interval) - for binary outcomes, presentation of both absolute and relative effect size | n/a – not a randomized trial and trial already reported |
| Harms | 27 | All harms or unintended events in each group | n/a – not a randomized trial and trial already reported |
| Ancillary analyses | 28 | Any other analyses performed, including subgroup and sensitivity analyses, distinguishing pre-specified from post-hoc | n/a – not a randomized trial and trial already reported |
| **Discussion** | | |  |
| Interpretation | 29 | Interpretation consistent with results, balancing benefits and harms, and considering other relevant evidence | n/a – not a randomized trial and trial already reported |
| Limitations | 30 | Trial limitations, addressing sources of potential bias, imprecision, generalisability, and, if relevant, multiplicity of analyses | n/a – not a randomized trial and trial already reported |

*We strongly recommend reading this statement in conjunction with the CONSORT 2025 Explanation and Elaboration and/or the CONSORT 2025 Expanded Checklist for important clarifications on all the items. We also recommend reading relevant CONSORT extensions. See [www.consort-spirit.org](http://www.consort-spirit.org).

Citation: Hopewell S, Chan AW, Collins GS, Hróbjartsson A, Moher D, Schulz KF, et al. CONSORT 2025 Statement: updated guideline for reporting randomised trials. BMJ. 2025; 388:e081123. <https://dx.doi.org/10.1136/bmj-2024-081123>.

© 2025 Hopewell et al. This is an Open Access article distributed under the terms of the Creative Commons Attribution License (<https://creativecommons.org/licenses/by/4.0/>), which permits unrestricted use, distribution, and reproduction in any medium, provided the original work is properly cited.
